# Oxidation Reactions Promoted or Catalyzed by Organotellurium Compounds

**DOI:** 10.1021/acsomega.5c03157

**Published:** 2025-07-03

**Authors:** Philipe Raphael O. Campos, Guilherme P. P. P. Silva, Yuga Shibuya, Shinichi Koguchi, Eduardo E. Alberto

**Affiliations:** † Departamento de Química, 28114 Universidade Federal de Minas Gerais, Belo Horizonte, MG 31270-901, Brazil; ‡ Department of Chemistry, 212250 Tokai University, 4-1-1 Kitakaname, Hiratsuka-shi, Kanagawa 259-1292, Japan

## Abstract

Oxidation of organic substrates is a pivotal transformation, with profound chemical and biological implications. This review covers the synthetic application of organotellurium compounds as promoters or activators of oxidizing agents in such reactions. This research field has evolved from utilizing stoichiometric amounts of organotellurium as oxidizing agents, producing copious amounts of side products, to using this class of compounds as catalysts. Another unique feature associated with using organotellurium compounds as catalysts for oxidation is the possibility of employing less hazardous oxidizing agents, such as peroxides or combinations of oxygen, photosensitizers, and light. These characteristics render the application of organotellurium compounds attractive from both an economic and environmental perspective.

## Graphical abstract

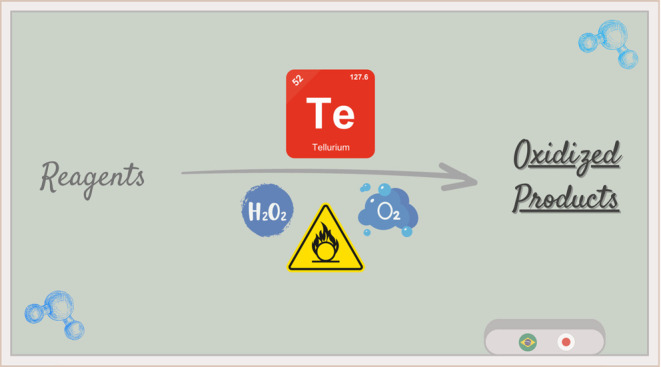

## Introduction

Oxidation reactions constitute fundamental processes with profound implications in all scientific fields. Among the chalcogen family, selenium-based compounds are pivotal in promoting oxidation reactions. The oxidation of organic substrates by selenium dioxide (SeO_2_) dates back almost a century.^1–3^ The interest in organoselenium compounds to promote (or activate) oxidizing agents rapidly grew after two major discoveries in the 1970s. The first one was the report that a-carbonyl-selenoxides **1** could deliver α,β-unsaturated carbonyl compounds **2**, upon heating (Scheme 1a).^4,5^ This transformation, also known as selenoxide syn elimination, represented a landmark in the utilization of organoselenium in oxidation reactions.

**Scheme 1:**
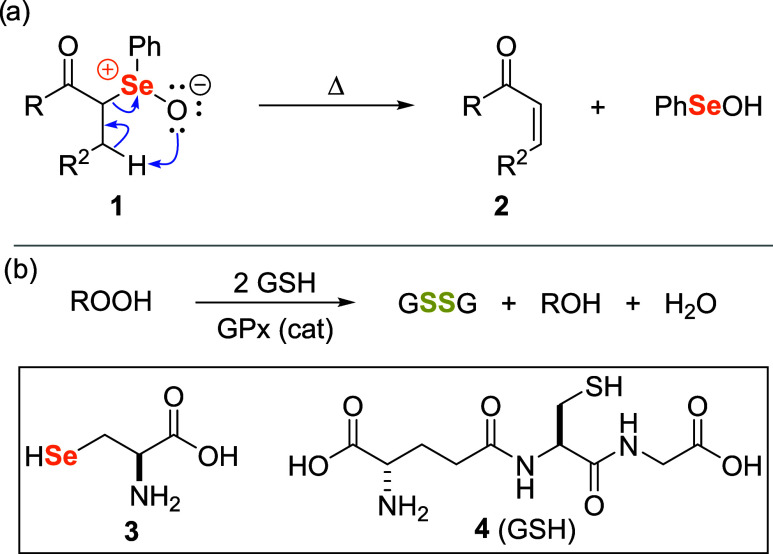
(a) Selenoxide syn Elimination Reaction. (b) Reduction of Peroxides Promoted by the Selenoenzyme Glutathione Peroxidase (GPx)

The second milestone was identifying that many mammals’ biochemical mechanisms were linked to selenium redox chemistry. Selenoenzymes constitute an important antioxidant system that protects biomembranes and other cellular components from oxidative stress.^6–9^ One of the most important is the enzyme glutathione peroxidase (GPx), which catalyzes the reduction of hazardous peroxides (ROOH) to water or alcohols (Scheme 1b).^10–12^ The most important amino acid in the active site of the enzyme is L-Selenocysteine **3**, which is responsible for reducing hydroperoxides at the expense of the tripeptide glutathione GSH **4**.^13,14^

The enormous popularity earned by organoselenium compounds certainly stimulated the research on organotellurium compounds.^15–18^ A growing interest in these compounds has been witnessed in past years due to their versatility and broad synthetic application.^19–26^ Our contribution highlights the progress in the application of organotellurium compounds in oxidation reactions.^27^ Whenever possible, a direct comparison between the similarities and differences in the chemical outcome of reactions compared to the selenium analogs will be provided. The current paper will be divided into two sections, the first discussing the aspects of oxidation with stoichiometric amounts of organotellurium compounds and the second for reactions carried out with catalytic amounts of organotellurium. The application of tellurium-based catalysts for the oxidation of thiols in the context of GPx-mimics will not be covered here, as extensive and up-to-date reviews have already covered this aspect.^28–31^

## Oxidation with Stoichiometric Amounts of Organotellurium

The first observation that organotellurium compounds could promote oxidation reactions was reported in 1977 by Wieber and Kaunzinger. It was found that dialkoxytellurides **5** react with 2 equiv of alcohols, producing **6** due to the alkoxide group substitution. However, the reaction of **5** with dithiols **7** occurred in a different pathway, resulting in the formation of the corresponding telluride **8** and disulfide **9** (Scheme 2).^32^

**Scheme 2:**
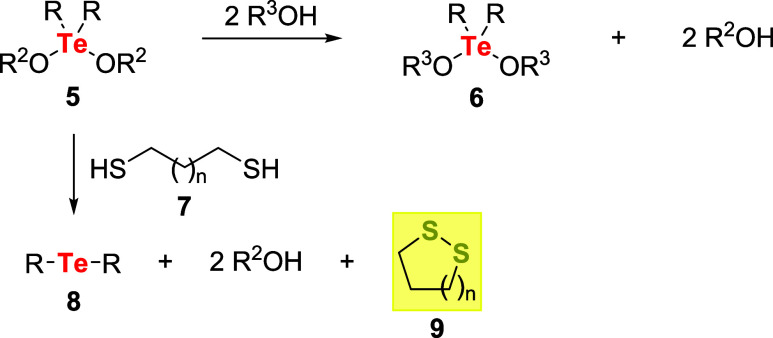
First Observation of an Organotellurium Compound Acting as an Oxidizing Agent

Following this finding, several research groups have dedicated efforts to studying the application of organotellurium compounds as oxidizing agents. Conversion of thio-compounds such as xanthates, thiocarbonates, thioamides, or thiones **10** into their corresponding oxo derivatives **11**, thiols **12** to disulfides **13**, phosphines **14** to phosphine oxides **15**, and dihydroxybenzene derivatives **16** to quinones **17** were the most common substrates employed for this purpose (Scheme 3).

**Scheme 3:**
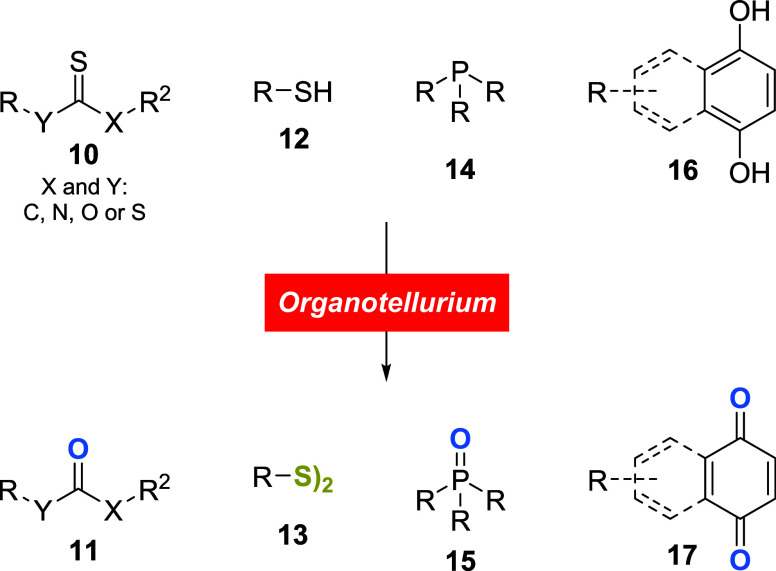
Substrates that are Commonly Used for Assessing the Oxidative Potential of Organotellurium Compounds

In 1979 Barton, Ley, and coauthors disclosed their findings on the use of bis­(*p*-methoxyphenyl)­telluroxide **18** as a mild and selective reagent for the conversion of **10** and **12** to products **11** and **13** as outlined on Scheme 4a.^33^ Telluroxide **18** which is odorless and stable in the dark at room temperature, was used in a stoichiometric amount (1.1 equiv related to the substrate) and the byproduct, telluride **19** could be isolated and reoxidized to **18** (the yield of **19** recovery was not disclosed).

**Scheme 4:**
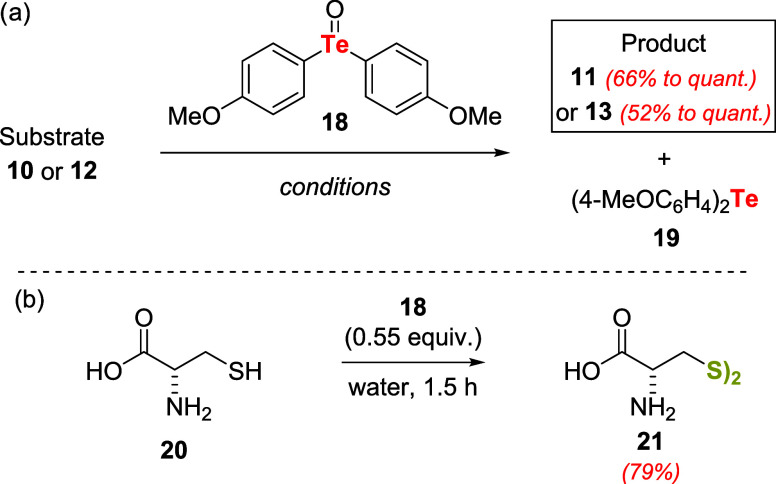
(a) Oxidation Reactions Using Telluroxide **18**. (b) Oxidation of l-Cysteine **20**, under Aqueous Conditions Using Telluroxide **18**

Among the thiols used as substrates, l-cysteine **20** was smoothly converted into the disulfide **21** using 0.55 equiv of **18**. In the case of **20**, water was the reaction solvent (Scheme 4b). Notably, other oxidizable functional groups, such as alcohols, enamines, and amines, were not affected by telluroxide **18**.

Subsequently, Cava and Engman introduced bis­(*p*-methoxyphenyl)­tellurone **22** as an oxidizing agent. Besides the known activity toward thiols **12** and dihydroxybenzene derivatives **16**, this reagent was also capable of promoting the conversion of benzylic alcohols **23** to the corresponding aldehyde or ketone **24** in high yields (Scheme 5).^34^

**Scheme 5:**
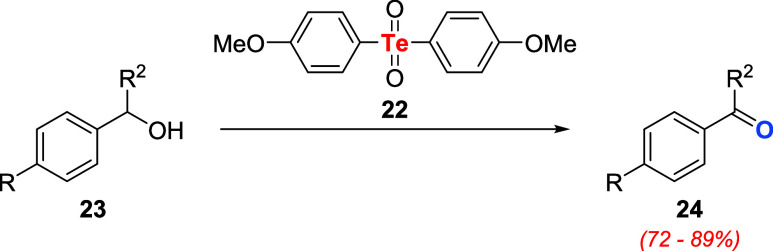
Oxidation of Benzylic Alcohols **23** Using Tellurone **22**

A few years later, Ogura et al. prepared polystyrene-bound selenoxide **25** and telluroxide **26** (Figure 1). These compounds effectively promoted the oxidation of dihydroxybenzenes **16**, thiols **12**, and phosphines **14** using solvents such as CH_2_Cl_2_, CHCl_3_, or AcOH.^35^ Regarding reactivity, the polymeric and monomeric species gave similar results; however, an important improvement was the possibility of recycling and reusing the polymeric reagent. Another interesting observation was that reactions with telluroxide **26** proceeded faster than those using selenoxide **25**.

**Figure 1:**
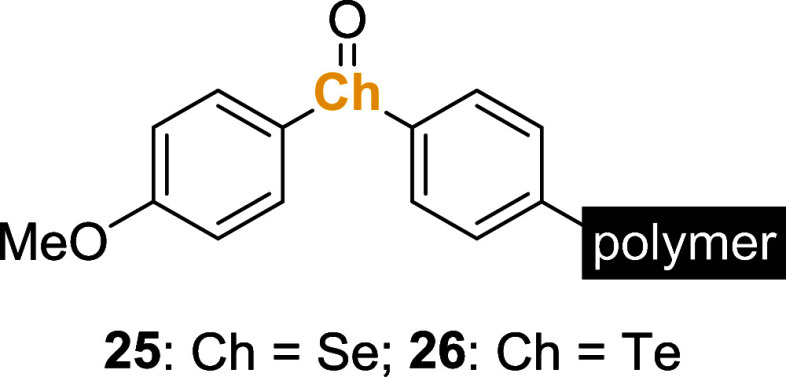
Polymer-tethered selenoxide **25** or telluroxide **26** was used for oxidation reactions.

In the same report, the authors studied the oxidation of thioamide **27** with telluroxide **26**. This reaction proved to be solvent-dependent: in acidic media (acetic acid as the solvent), the main products were 1,2,4-thiadiazoles **28**, and in other solvents (e.g., EtOH, CH_2_Cl_2_), nitriles **29** were obtained.

The formation of products **28** and **29** was affected by adding the thioamide **27** to the telluroxide **26**, leading to adduct **30**. Under neutral conditions, **30** degrades spontaneously to nitrile **29** and telluride (Scheme 6b). Conversely, under acidic conditions, **30** could react with another 1 equiv of thioamide **27** to give a termolecular intermediate **31**. The collapse of **31** led to the dimerization product 1,2,4-thiadiazole **28** and telluride (Scheme 6c).

**Scheme 6:**
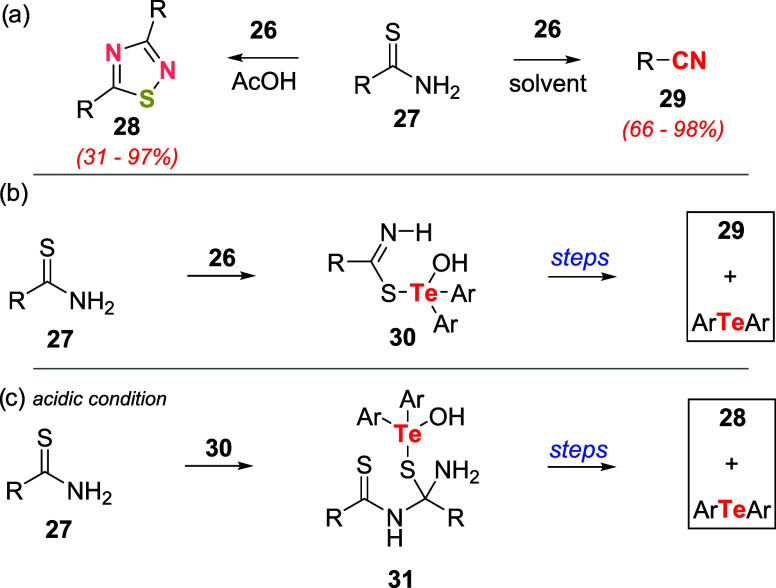
(a) Oxidation of Thioamides **27** Using **26**. (b) Formation of Nitriles **29** under Basic Conditions. (c) Formation of the Dimer 1,2,4-thiadiazole **28** under Acidic Conditions

In the same year, Barton and coauthors introduced tellurinic acid anhydrides **32a**–**c** as oxidizing agents (Figure 2). These compounds are sparingly soluble in common organic solvents, except in AcOH. Similar to the previous studies, it was observed that phenols were inert to **32a**–**c**, and dihydroxybenzene derivatives **16** and thiols **12** could be readily converted to quinones **17** and disulfides **13**, respectively.^36^

**Figure 2:**
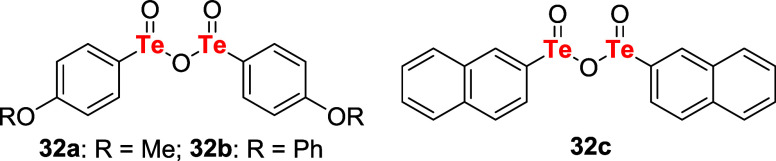
Tellurinic acid anhydrides **32a**–**c** were studied as oxidizing agents.

Competition reactions were designed to compare the relative reactivities of tellurium compounds. Using dihydroxybenzene as substrate, it was found that telluroxide **18** was a slightly better oxidant agent compared with the tellurinic acid anhydride **32a**. Within **32a**–**c**, the 2-naphthalene derivative **32c** was the best oxidizing agent and **32b** the worst. Interestingly, blank experiments containing only organotellurium compounds showed that telluride **19** is oxidized to telluroxide **18** by tellurinic anhydride **32b**. However, no reaction occurred between telluroxide **18** and ditelluride **33** (Scheme 7).

**Scheme 7:**
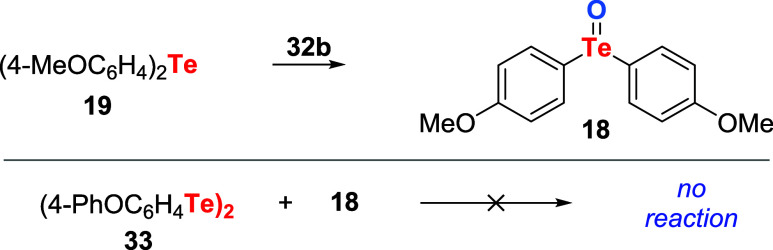
Evaluation of the Capacity of Tellurinic Anhydride and Telluroxide to Oxidize Organotellurium Compounds

The same group introduced (*p*-methoxyphenyl)-morpholiniumtellurium­(IV) dichloride **34** as an oxidizing agent (Figure 3).^37^ **34** can be readily prepared from (*p*-methoxyphenyl)tellurium trichloride and *N*-trimethylsilyl morpholine and promotes the oxidation of dihydroxybenzene and derivatives in the presence of pyridine. Compound **34** showed higher activity when compared to the corresponding tellurinic acid anhydride **32a** and was comparable to telluroxide **18**. Noteworthy, the *bis*-morpholine derivative **35** outperformed all of the other reagents examined. Oxidation reactions with **35** produced elemental tellurium and morpholine hydrochloride as side products.

**Figure 3:**
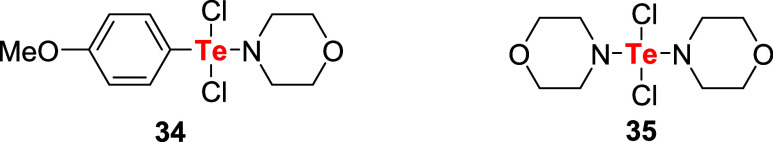
Tellurium­(IV) derivatives **34** and **35** were employed as oxidizing agents.

Kambe, Sonoda, and coauthors disclosed that tellurinic acid anhydride **32d** is reduced to diphenyl ditelluride **36**, accompanied by the conversion of styrene **37** to diacetate **38** in refluxing AcOH (Scheme 8). Using a catalytic amount of H_2_SO_4_, the reaction yield could be substantially increased.^38^

**Scheme 8:**
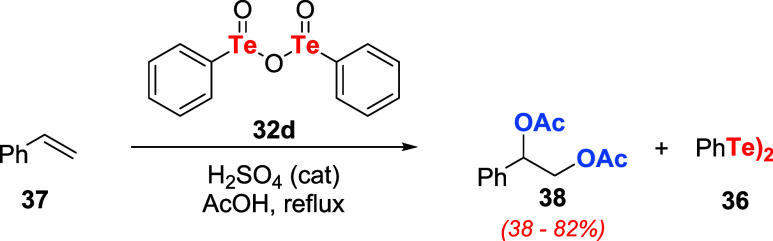
Oxidation of Styrene **37** to the Corresponding Diacetate **38** Using Tellurinic Acid Anhydride **32d**

It was observed that terminal alkenes react faster than internal ones and that the reaction had an induction period of about 5 h. Adding a reducing agent such as **36** (PhTeTePh) significantly accelerated the reaction rate. It was postulated that the active oxidizing agent was tellurenic acid **39**, produced *in situ* from tellurinic acid anhydride **32d** and PhTeTePh **36**. The final product **38** was formed after the acid hydrolysis of telluride **40** (Scheme 9). Control experiments corroborate the formation of **40** as a reaction intermediate.

**Scheme 9:**
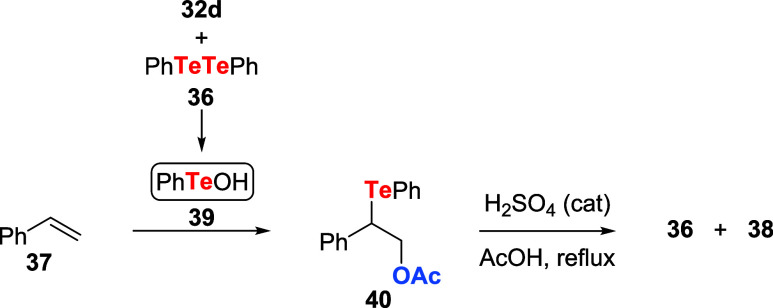
Formation of Tellurenic Acid **39**, the Active Species on Oxidation with Tellurinic Acid Anhydride **32d** and PhTeTePh **36**

Independently, Ogura and coauthors showed the usefulness of tellurinic acid anhydrides **32** in oxidation reactions. It was identified that tellurenil acetate **41** is the active oxidizing agent for transformations employing **32** in AcOH or acetic anhydride under reflux (Scheme 10a). Among other substrates, **41** promoted the conversion of unsaturated alcohols **42** to the corresponding cyclic ether telluride **43**. The reaction showed high regio- and stereoselectivity in refluxing AcOH following reduction with hydrazine (Scheme 10b).^39^ Under appropriate conditions, detelluration of **43** allowed the preparation of other products (e.g., alkenes, bromides, and ethers **44**).^40^

**Scheme 10:**
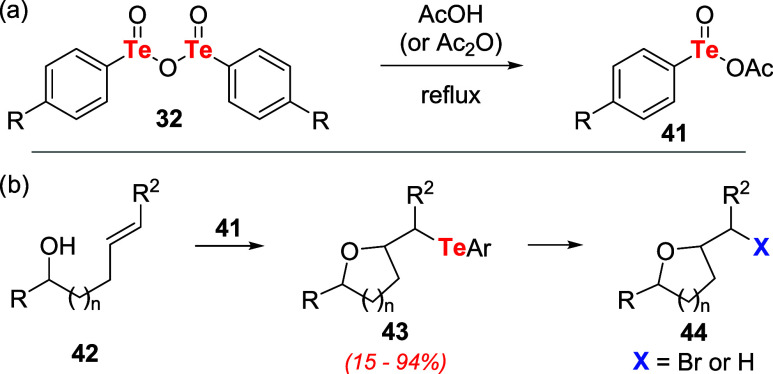
(a) Preparation of Tellurenil Acetates **41** from Tellurinic Acid Anhydrides **32** and Acetic Acid. (b) Conversion of Unsaturated Alcohols **42** to Functionalized Cyclic Ether **44**

In the same report, the authors disclosed aminotelluration of alkenes **45** using benzenetellurinyl acetate **41**, excess ethyl carbamate, and a Lewis acid (Scheme 11).^41,42^ At room temperature, the main product obtained was telluride **46**, and at higher temperatures (refluxing 1,2-dichloroethane, for instance), 2-oxazolidines **47** were obtained in high yields, accompanied by the corresponding ditelluride.

**Scheme 11:**
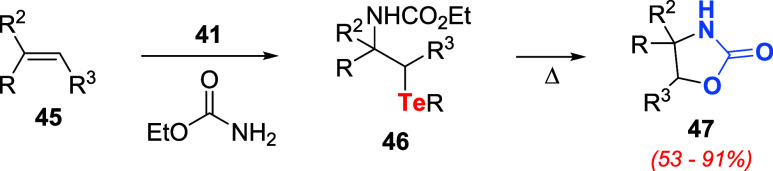
Aminotelluration of Alkenes to Produce 2-Oxazolidines **47**

Following these findings, the same group studied the oxidation ability of mixed anhydrides **48a**–**c**, prepared from tellurinic acid anhydride **32** and the corresponding acid anhydride (Figure 4). All of them efficiently promoted the oxidation of benzenethiol and triphenilphosphine. For α-hydroxy ketones as substrates, mixed anhydride **48b** showed better activity. On the other hand, the products’ selectivity, observed by the oxidation of thioamides or thioureas, depended on the reagent used. While **48a** gave exclusively nitriles **29**, **48c** produced predominantly 1,2,4-thiadiazoles **28**.^42^

**Figure 4:**
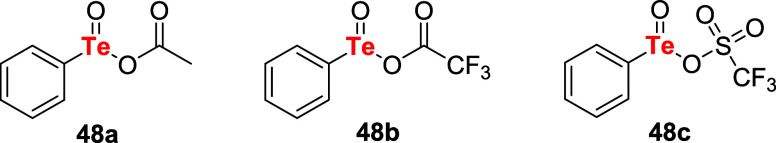
Tellurinic acid mixed anhydrides **48a**–**c** were used as oxidizing agents.

Since the discovery of the oxidative potential of telluroxides, it has been known that they do not promote the oxidation of alcohols.^33,35^ Considering that telluroxides can reversibly form aggregates held together by tellurium–oxygen interactions,^43,44^ and that they can react with water to produce hydrates,^45,46^ Nishiyama, Ando, and coauthors reasoned that these characteristics might be hampering the utilization of telluroxides as oxidizing agents. To prevent aggregate formation, they proposed utilizing bulky telluroxides **49a**–**b** as catalysts (Scheme 12).^47^ Interestingly, a combination of molecular oxygen, light, and a photosensitizer was introduced as an alternative to preparing telluroxides. Under these conditions, singlet oxygen is generated, allowing for the formation of telluroxides in quantitative yields in a short reaction time.

**Scheme 12:**
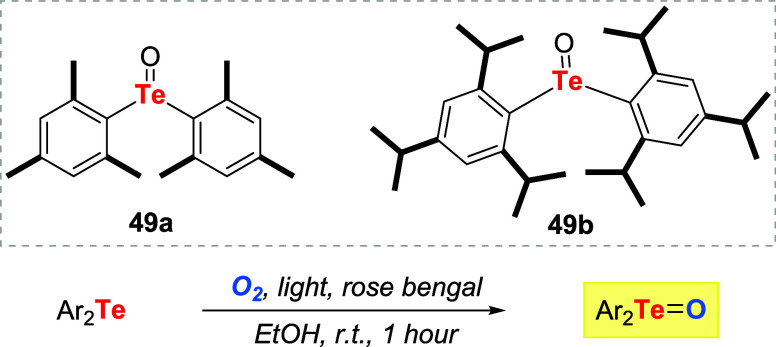
Conversion of Tellurides to Telluroxides Promoted by Oxygen, Light, and Photosensitizer

Oxidation of allylic and benzyl alcohols **23** under azeotropic conditions (refluxing *p*-xylene) showed that bulky telluroxides **49a** and **49b** were about six times more effective as oxidizing agents when compared to less hindered telluroxide **18**. The reaction scope included benzylic alcohols having electron-donating and electron-withdrawing groups and allylic alcohols (Scheme 13a). Unfortunately, nonactivated alcohols such as 1-octanol were not oxidized. The proposed mechanism involves the nucleophilic addition of alcohol to telluroxide, producing adduct **50**. Then, intermediate **50** collapses, delivering the desired carbonyl compound **24**, water, and diaryl telluride (Scheme 13b).

**Scheme 13:**
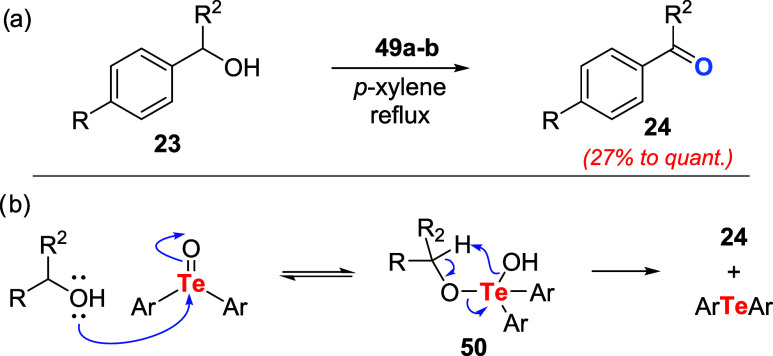
(a) Oxidation of Alcohols to Aldehydes or Ketones by Telluroxides **49a**–**b**. (b) Proposed Reaction Mechanism

The first example of the oxidation of a primary nonactivated alcohol, such as 1-dodecanol, was reported later by the same group. For this task, 2.0 equiv of the bulky tellurone **51** were employed as an oxidant (Scheme 14).^48^

**Scheme 14:**
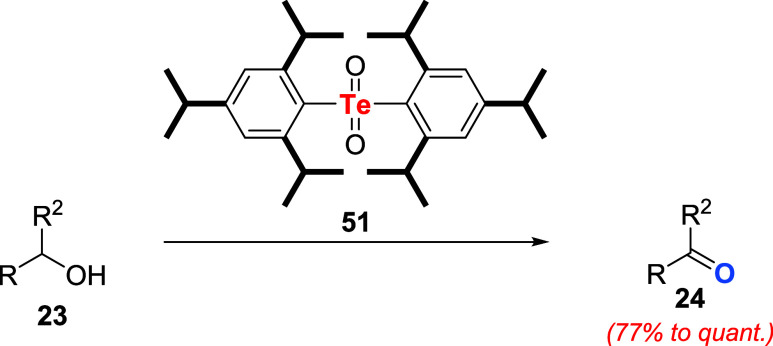
Oxidation of Alcohols to Aldehydes or Ketones by Tellurone **51**

In most experiments, benzylic alcohols **23** were smoothly converted to aldehydes or ketones **24** in quantitative yields. On the other hand, nonactivated secondary alcohols proved to be challenging substrates. Noteworthy tellurone **51** afforded the desired products under milder reaction conditions compared to analogous telluroxide **49b**, indicating that the former is a better oxidizing agent.

Recently, Koguchi et al. disclosed that diaryl tellurides react with the appropriate acid under aerobic conditions upon irradiation with a white light-emitting diode (LED) and as little as 0.01 mol % of a photosensitizer such as tetraphenylporphyrin (TPP) to produce diaryltellurium dicarboxylates **52** in quantitative yields. The formation of diaryltellurium dicarboxylates **52** is proposed to proceed via photooxidation to the corresponding tellurides (Scheme 15a). Initially, triplet oxygen is converted to singlet oxygen by the photosensitizer under visible light irradiation. Subsequent singlet-oxygen oxidation of the telluride affords the corresponding telluroxide, which then reacts with 2 equiv of carboxylic acid to furnish the diaryltellurium dicarboxylates **52** and water. These compounds could oxidize benzoin (3-hydroxy-1,3-diphenylpropan-1-one) and derivatives **53** to **54** in excellent yields. Among the diaryltellurium dicarboxylates tested, compounds derived from trifluoroacetic acid were the most active (Scheme 15).^49^

**Scheme 15:**
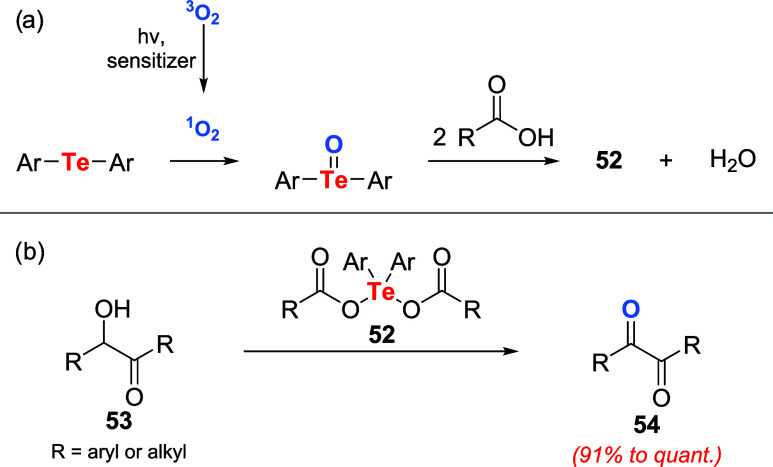
(a) Proposed Mechanism for the Formation of Diaryltellurium Dicarboxylates **52**. (b) Utilization of Diaryltellurium Dicarboxylates **52** for the Oxidation of Benzoin Derivatives **53**

## Oxidation with Catalytic Amounts of Organotellurium

The first example of a catalytic oxidative process employing organotellurium compounds was disclosed by Ley, Barton, and Meerholz, early in 1980.^50,51^ Thiocarbonyl derivatives **10** were converted to the corresponding carbonyls **11** using as little as 1.5 mol % of tellurides **19** or **56a**–**b** (Scheme 16). For this purpose, an excess of 1,2-dibromotetrachloroethane **57** was used as a brominating agent, converting **19** or **56a**–**b** to dibromide **58a**–**c**, which was promptly converted to telluroxide **18** or **55a**–**b** in aqueous potassium carbonate. They also found that reactions using a more electron-rich telluride, such as the bis-dimethylamino **56a**, were faster and that the water-soluble telluride **56c** was less reactive than **19**, respectively.

**Scheme 16:**
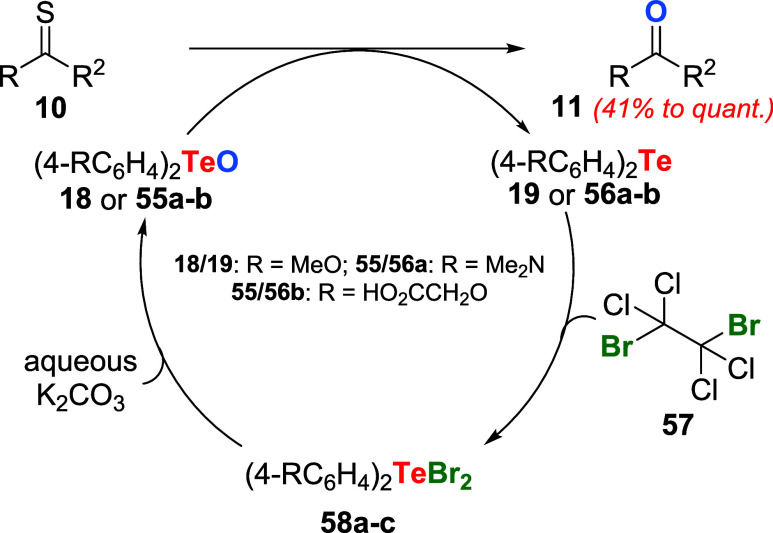
First Example of an Oxidation Reaction Using a Catalytic Amount of Organotellurium

On the other hand, in continuation of their studies on the application of tellurinic anhydrides as oxidizing agents in organic transformations,^39–42^ Ogura and coauthors observed that **32a** was capable of selectively catalyzing the hydration of terminal alkynes **59** to ketones **60** in refluxing acetic acid (Scheme 17).^52^

**Scheme 17:**
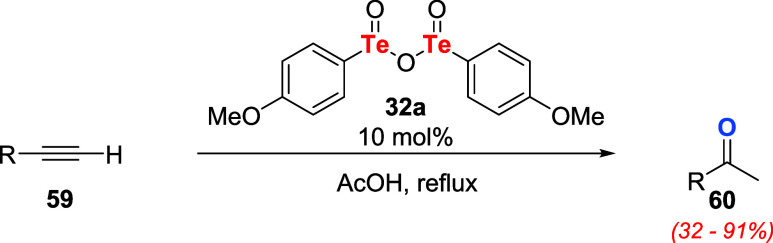
Tellurinic Anhydride **32a** as a Catalyst for the Hydration of Terminal Alkynes **59**

A few years later, the same group introduced the synthesis of bis­(*p*-methoxyphenyl)tellurium dicarboxylates **61** or the corresponding telluroxide hydrate **62** by electrolysis of **19**. The reaction was performed with tetrabutylammonium acetate as an electrolyte in platinum electrodes. The reaction media could define product selectivity. In dry acetonitrile, **61** was obtained, and in wet MeCN, **62** was the main product (Scheme 18).^53^

**Scheme 18:**
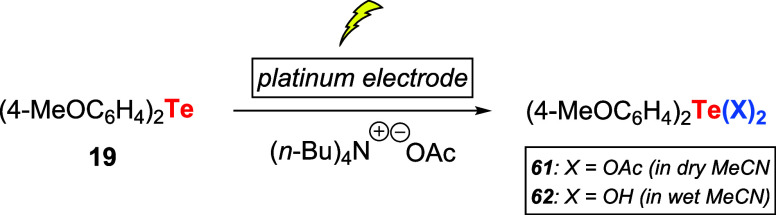
Synthesis of Bis­(*p*-methoxyphenyl)­tellurium Dicarboxylates **61** or **62** under Electrolysis Conditions

This reaction setup was employed to promote the oxidation of thioamides **27** to the corresponding nitriles, **29** or 1,2,4-thiadiazoles **28**, using 5.0 mol % of telluride **19**. The former product was obtained preferably under anhydrous conditions and the latter in aqueous solutions.

Another interesting environmental option for oxidation reactions is hydrogen peroxide, which is a strong, inexpensive, commercially available oxidizing agent that is stable in aqueous solutions. Another great advantage of hydrogen peroxide is that upon decomposition it produces only water and oxygen. However, although a strong oxidizing agent, kinetically, H_2_O_2_ oxidations can be slow and often require a catalyst to accelerate the reactions to synthetically useful rates. Accordingly, Brill studied the application of arenetellurinic acid anchored to a divinylbenzene-styrene copolymer **63** as a catalyst to promote the epoxidation of alkenes **45** using hydrogen peroxide as an oxidizing agent. The postulated active species was the arenepertellurinic acid **64** (Scheme 19).^54^

**Scheme 19:**
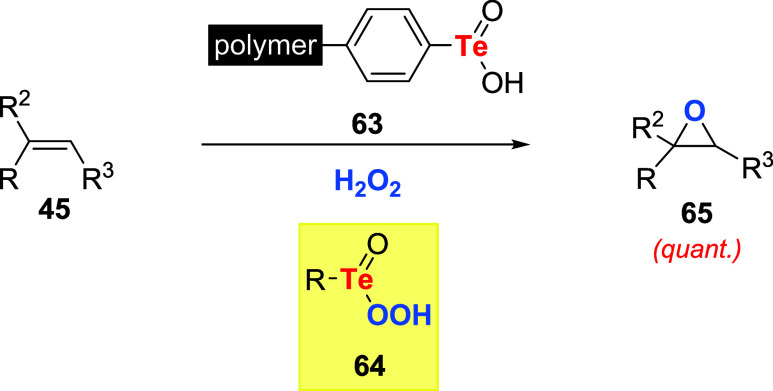
Polymer-Tethered Tellurinic **63** as a Catalyst for Epoxidations Using H_2_O_2_

It was observed that alkenes **45** (except for allyl alcohols) could be stereospecifically and quantitatively converted to epoxides **65**. Reaction rates were modulated by the nature of alkenes, which are the electron-rich substrates with the fastest speeds. Additionally, catalysts prepared from the most highly cross-linked resins were the most active. Interestingly, discrete arenetellurinic acids showed no catalytic activity. It was reasoned that the lack of catalytic activity of these tellurinic acids was due to the formation of tellurinic acid anhydrides **32**. However, when oxidations were conducted with polymeric catalyst **63** in the presence of added tellurinic acid, no diminution of the reaction rate occurred. Moreover, a polymer prepared with randomly placed tellurinic acid displayed little activity. The author concluded that these observations represent an example where the steric effect of a rigid support lowers the entropy of activation and enhances the formation of what would in solution be the least favored active form of the catalyst.

Following their initial findings on the oxidation of alkenes with tellurinic acids **32** and derivatives,^38^ Kambe, Sonoda, and coauthors disclosed that it was possible to reach the desired products using a catalytic amount of PhTeTePh **36** and an oxidizing agent (Scheme 20).^55,56^ Activation of *t*-BuOOH with 10 mol % of **36**, related to **37**, allowed the preparation of compound **38** in 88% yield, albeit the long reaction time required (3.8 days). Using a 50.0 mol % load of **36**, H_2_O_2_, or O_2_ gave comparable results to *t*-BuOOH, although the reaction rate using O_2_ was slower.

**Scheme 20:**
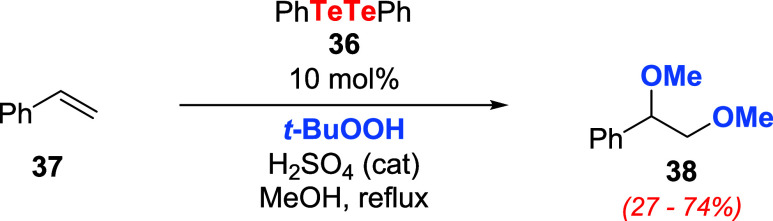
Oxidation of Styrene **37** with *t*-BuOOH and 10 mol % of Ditelluride **36**

On the other hand, Detty and coauthor prepared the dihydroxytellurane **67** derived from the tellurapyrylium dye **66** and evaluated its application in oxidation reactions. **67** could be produced from **66** by the oxidative addition of hydrogen peroxide or by a self-sensitized singlet-oxygen scavenging process (Scheme 21a).^57,58^ Synthetically, the oxidative addition of H_2_O_2_ across tellurium to produce **67** was fast, with a second-order rate constant of about 8.0 × 10^8^ M^–1^ s^–1^. Moreover, dihydroxytellurane **67** could oxidize substrates such as thiols **12** or leuco dyes **68** faster than hydrogen peroxide (Scheme 21b). Collectively, these aspects allowed the application of **66** as a catalytic activator for H_2_O_2_, delivering the oxidized product **69** in about 4 orders of magnitude faster than in the control experiment (absence of **66**). Additionally, photochemical oxidation of **68** in the presence of **66** in air-saturated solutions via light irradiation delivered **69** with turnover numbers above 70.

**Scheme 21:**
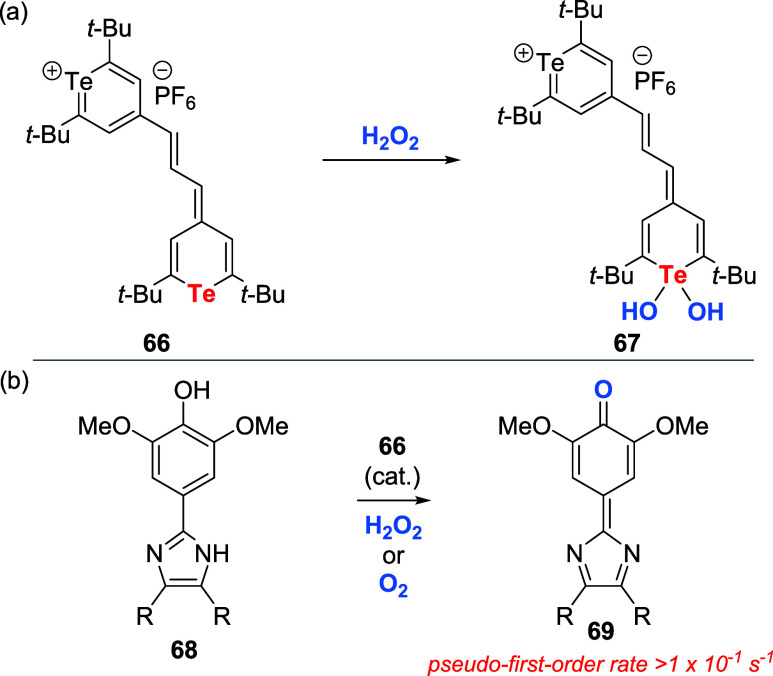
(a) Conversion of Tellurapyrylium Dye **66** to the Corresponding Dihydroxytellurane **67** with H_2_O_2_. (b) Oxidation of Dyes Promoted by **66**

In the following years, Detty′s group developed a series of organotellurium compounds and evaluated them as catalysts for the activation of H_2_O_2_ in the oxidation of halide salts (Scheme 22). In this reaction, the positively charged halide ion is trapped by an appropriate substrate to produce a halogenated product.

**Scheme 22:**
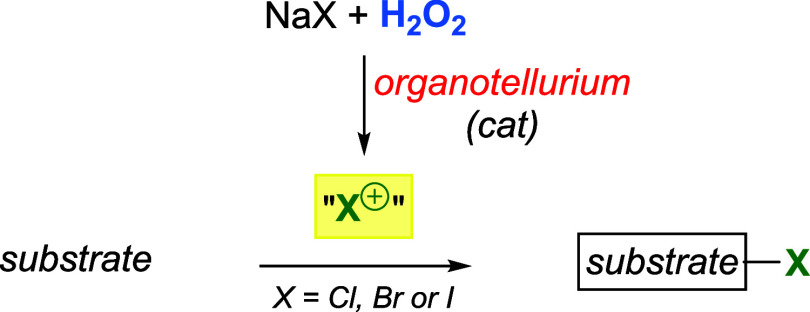
Organotellurium as Catalytic Activators of H_2_O_2_ for the Oxidation of Halide Salts and Subsequent Halogenation of Organic Substrates

For instance, the oxidation of sodium bromide with hydrogen peroxide was followed by the initial rate of formation of dibromide **71** and bromohydrin **72** in a two-phase system of cyclohexene **70** (used in excess), H_2_O_2_, NaBr, CH_2_Cl_2_, and pH 6.0 phosphate buffer (Scheme 23).^59^ Rates of control experiments were compared to reactions catalyzed by **66**, **73**, or water-soluble **74** (0.1 mol % relative to cyclohexene). Catalyst **73** was the most active, and **66** was the least active. Reactions with **73** and **66** were, respectively, 42 and 9 times faster than those in the control experiment. Oxidation of sodium chloride by H_2_O_2_ with these catalysts was observed, but the process was sluggish.

**Scheme 23:**
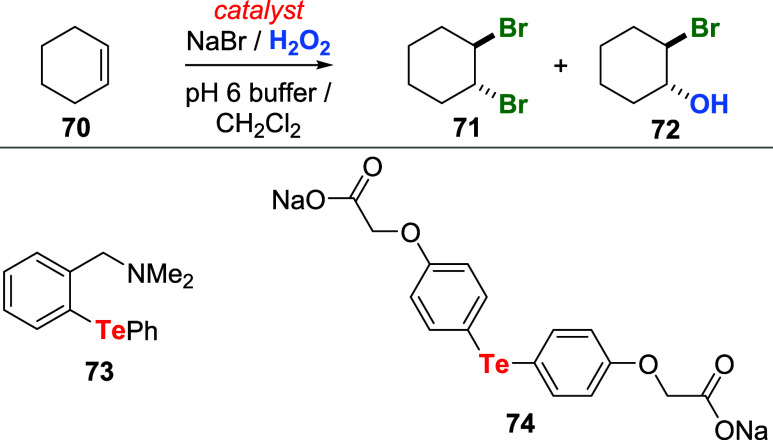
Bromination of Cyclohexene Using Tellurides, NaBr, and H_2_O_2_

Compounds such as the dendrimer **75**, water-soluble telluride **76**, and telluraporphyrin **77** were also efficiently used as catalysts for the activation of H_2_O_2_ toward oxidation of halide salts (Figure 5). The dendrimer catalyst **75**, with 12 reactive “PhTe” units, promoted the conversion of cyclohexene **70** to dibromide **71** and bromohydrin **72** in a two-phase system. The catalytic activity of this sort of compound was directly proportional to the number of “PhTe” units.^60^

**Figure 5:**
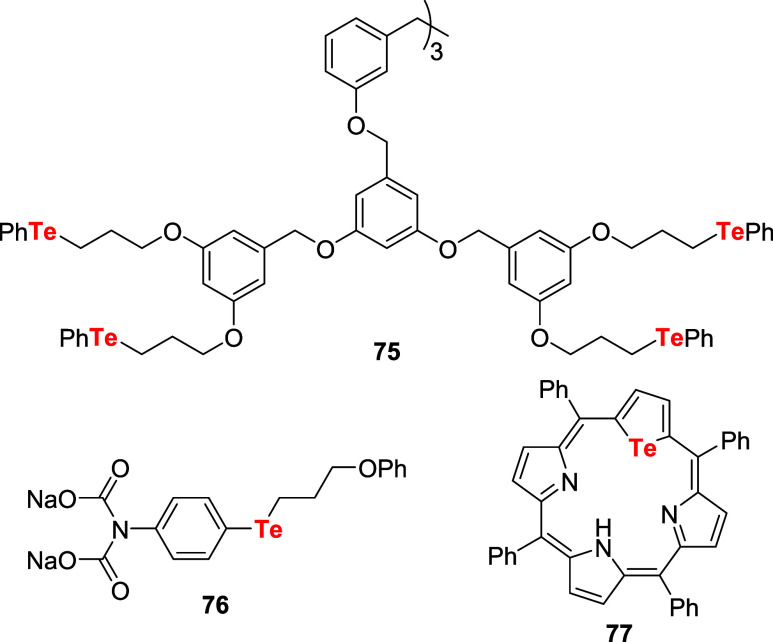
Structures of organotellurium catalysts employed for the activation of H_2_O_2_.

On the other hand, telluride **76** was designed to provide water solubility and accelerate its oxidation with H_2_O_2_ due to the increase of electron density on tellurium promoted by the amino substituent. Moreover, the oxygen atom of the phenoxypropyl group could chelate to the oxidized tellurium center via a five-membered ring. Oxidation of halide salts (iodide and bromide) with H_2_O_2_ was performed under two-phase conditions using **76** as a catalyst. Unsaturated acids **78** and activated aromatic compounds **80** were used to monitor the progress of the reaction (Scheme 24).^61^

**Scheme 24:**
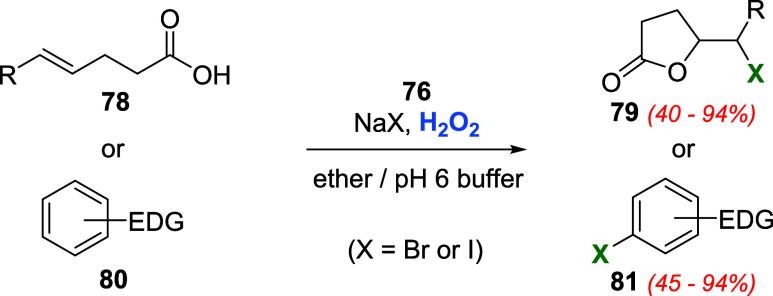
Synthesis of Halolactones **79** and Aryl Halides **81** by Oxidation of Halide Salts with H_2_O_2_ Catalyzed by Telluride **76**

For iodination reactions, 0.8 mol % of catalyst **76** (related to the substrate) promoted the formation of products **79** or **81** in a range of 40 to 94% yield. Less than 10% of substrate conversion was observed without the catalyst under the same reaction conditions. On the other hand, bromination required higher concentrations of bromide, peroxide, catalyst **76** (2.5 mol %), and longer reaction times. A better outcome for the bromide ion oxidation with H_2_O_2_ was achieved using telluraporphyrin **77** as a catalyst. The addition of 0.2 mol % of telluraporphyrin **77** (relative to 4-pentenoic acid **78**, R=H) accelerated the reaction compared to the control (23-fold increase in the rate relative to the uncatalyzed reaction). In preparative experiments, 4-pentenoic acid was converted to the corresponding bromolactone **79** in 94% yield in a CH_2_Cl_2_ and pH 6 phosphate buffer two-phase system.^62^

Later, diorganotellurides bearing triethoxysilane functionalities were generated *in situ* and incorporated into tetraethoxysilane (TEOS) xerogel monoliths **82** and **83a**–**d**. The final concentration of the catalyst was 2.5 mol % relative to TEOS (Scheme 25). These catalysts tethered to a solid support were evaluated as hydrogen peroxide activators in the oxidation of NaBr. For these experiments, the load of **82** and **83a**–**d** was 2.0 mol % relative to the substrate: 4-pentenoic acid **78** (R=H) dissolved in a pH 6 phosphate buffer.^63^

**Scheme 25:**
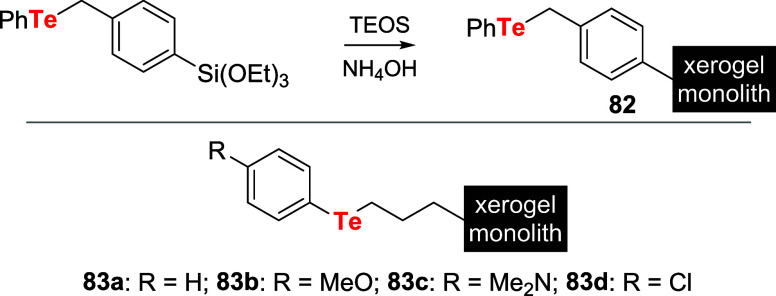
Preparation of Tellurides Anchored into Solid Support

Among all test catalysts, benzylic telluride **82** showed the best activity. Among tellurides **83a**–**d**, catalyst **83c** assembled with the stronger electron-donating group was the most active. Nevertheless, no clear trend was observed considering the electron density on tellurium and catalytic activity. Catalyst **83a**, for instance, was about three times more active than catalyst **83b**. Another key factor was the recyclability of these catalysts. In this sense, although **82** was the most active, it did not recycle well. The first recycling showed only 20% of the initial reactivity, suggesting that the chalcogen atom did not remain sequestered in the xerogel throughout the reaction. Conversely, telluride **83a** showed good catalytic activity, and its rate of bromination remained unchanged after four cycles of reaction (approximately 2000 turnovers). Noteworthy, direct comparisons were made between tellurium and selenium catalysts. In all cases, tellurium analogs were more efficient for H_2_O_2_ activation.

The reaction mechanism for halide oxidation by H_2_O_2_ activated by tellurides is presented in Scheme 26. It is a multistep process involving oxidative addition, ligand exchange, and reductive elimination. Based on kinetic experiments, Detty et al. proposed that the rate-limiting step was either the reductive elimination or the cleavage of the halogen-tellurium bond. In other words, there is oxidation of the halide salt. The initial step postulated is telluride **8** oxidation to the corresponding telluroxide **84**. Telluroxide could be added to water in an aqueous solution to form dihydroxytellurane **85**. Subsequent nucleophilic attack by the halide anion at the ligated hydroxide oxygen of **85** would give telluride **8** and hypohalous acid (a halogenating agent). After the initial oxidation of **8** with H_2_O_2_, another plausible route involves the addition of a halide to telluroxide **84**, producing the halotelluronium salt **86**. This intermediate can either suffer a nucleophilic attack of another halide, producing X_2_, regenerating the telluride **8** to re-enter the catalytic cycle, or lose water, producing **87**. The latter species can also react with halide, delivering molecular halogen and telluride **8**. Catalysts assembled with chelating groups may show a less complex mechanism. Upon oxidation with H_2_O_2_, they produce a chelated intermediate such as **88**. This architecture would prevent the formation of dihydroxytellurane **85** and other intermediates. Besides, a direct attack of halide at the ligated hydroxide oxygen can be anticipated, producing HOX. Additionally, the chelating ligand prevents the addition of a second halide to the oxidized tellurium atom, such as **87**, producing R_2_TeX_2_ adducts.^64–67^

**Scheme 26:**
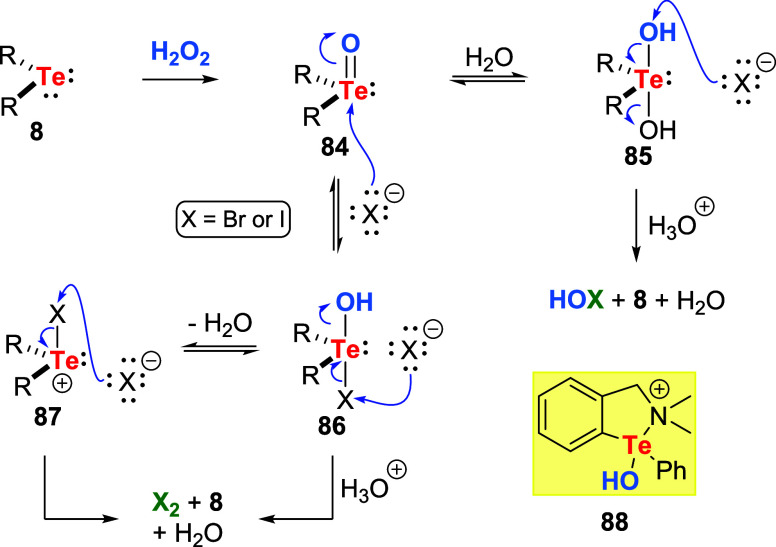
Proposed Reaction Mechanism for the Activation of H_2_O_2_ by Tellurides **8** in the Oxidation of Halide Salts

Detty’s group also explored the utilization of diaryl ditellurides as catalysts to promote hydrogen peroxide activation. Several catalysts **36** or **89a**–**g** in which tellurium experiments different electronic environments, interaction with a chelating group, or is sterically encumbered were tested for the purpose (Figure 6).^68^

**Figure 6:**
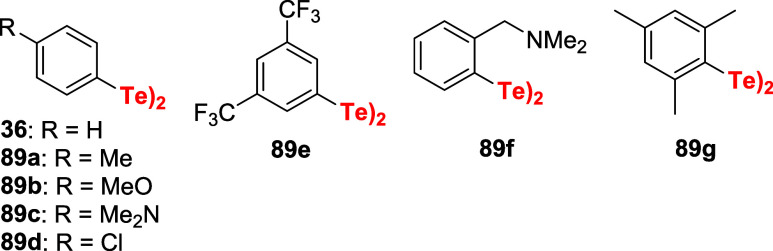
Structures of diaryl ditellurides studied as catalytic activators of H_2_O_2_.

The oxidation of NaBr with H_2_O_2_ in buffered aqueous solutions was monitored by observing the consumption of 4-pentanoic acid **78** (R=H) using 0.2 mol % of ditellurides **36** or **89a**–**g** as catalysts (relative to **78**). Surprisingly, the most active catalyst was the simpler one: diphenyl ditelluride **36**, which promoted a 240-fold increase in the reaction rate. Kinetic experiments revealed that ditellurides possessing electron-donating groups **89a**–**c** were efficient catalysts, with the catalytic activity being inversely proportional to the donating capacity of the substituent. Ditelluride **89f** with a pendant chelating group also showed catalytic activity. Conversely, sterically hindered ditelluride **89g** and ditellurides containing electron-withdrawing groups **89d**–**e** were inactive.

Control experiments were designed to identify the active oxidant in the catalytic cycle. It was observed that after the first reaction cycle, the ditelluride **89b** or species produced from it were no longer functional as catalysts. Accordingly, the reaction of **89b** with varying amounts of H_2_O_2_ was examined using ^125^Te NMR spectroscopy in which tellurinic acid **90b** (presumably in equilibrium with its anhydride derivative **91b**), and telluronic acid **92b** were identified as oxidized intermediates produced by **89b** and H_2_O_2_ (Scheme 27).

**Scheme 27:**
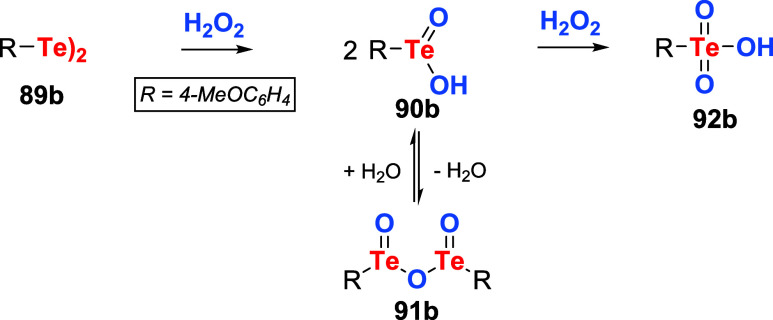
Observed Products of Ditelluride **89b** Oxidation with H_2_O_2_

Then, the catalytic activity of authentic samples of tellurinic acid **90b** and telluronic acid **92b** was examined in the oxidation of NaBr with H_2_O_2_. It was found that tellurinic acid **90b** and ditelluride **89b** gave comparable rates of bromination, but telluronic acid **92b** was inactive. Control experiments using **90b** in a stoichiometric amount revealed that it was not an oxidizing agent. Nonetheless, **90b** promoted oxidation when combined with hydrogen peroxide. Collectively, these results allowed the formulation of the reaction mechanism for the bromide salt oxidation using H_2_O_2_ catalyzed by diaryl ditellurides. The initial step is the oxidation of ditelluride **89b** to corresponding tellurinic acid **90b**. Depending on the nature of the catalyst and the concentration of peroxide, **90b** can be further oxidized to **92b**, representing catalyst inactivation. On the other hand, the catalytic process is possible due to the equilibrium between **90b** and **93b** in acidic media, followed by the addition of H_2_O_2_ to **93b**, producing the active species pertellurinic acid **94b** (Scheme 28).

**Scheme 28:**
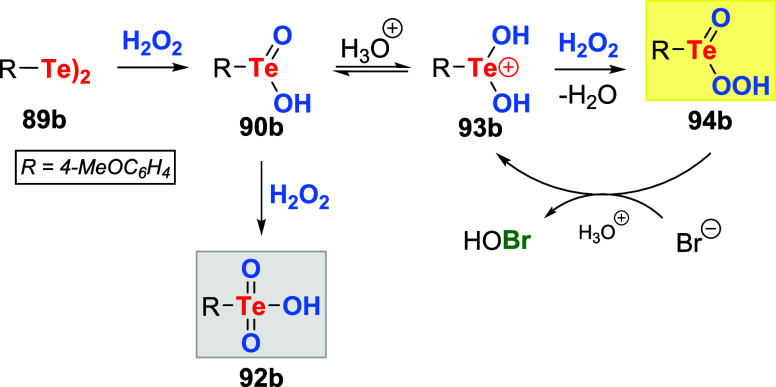
Proposed Reaction Mechanism for H_2_O_2_ Activation by Ditelluride **89b** in the Oxidation of Halide Salts

Following this report, Alberto and Martins explored the broadening of the synthetic utility of bromination reactions utilizing PhTeTePh **36** as a catalyst combined with NaBr and H_2_O_2_.^69^ Albeit water-soluble or highly reactive substrates such as 4-pentenoic acid **78** (R=H) and 1,3,5-trimethoxybenzene **95** could be effectively converted to products **79** or **96**, an attempt to use the reported conditions^68^ for the dibromination of cyclohexene **70** produced **71** in only 14% yield (Scheme 29).

**Scheme 29:**
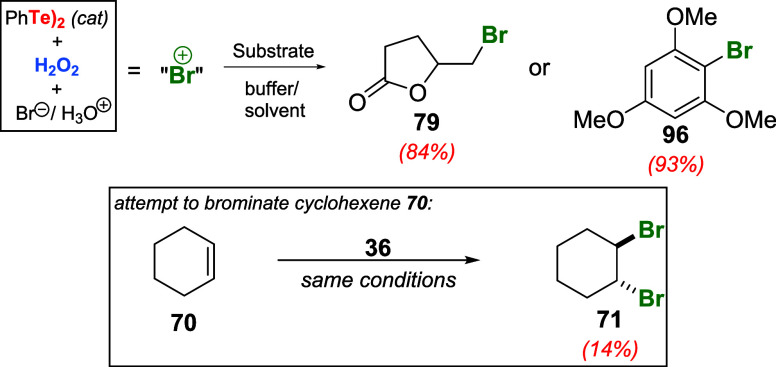
Attempt to Produce 1,2-dibromocyclohexene **71** Utilizing PhTeTePh **36** as a Catalyst Combined with NaBr and H_2_O_2_ in Aqueous Media

The authors reasoned that the mass transfer of “Br^+^” species in aqueous solutions was hampering the bromination of “simple” alkenes with H_2_O_2_/bromide due to the hydrophobic nature of the substrate. It becomes a problem as “Br^+^” species generated in the aqueous layer promote the irreversible disproportionation of H_2_O_2_ to oxygen and water.^70^ The destruction of H_2_O_2_ can be mitigated by acidifying the solution, as observed using aqueous buffered solutions. Accordingly, acetic acid was used as a cosolvent to efficiently promote the bromination of nonactivated (and hydrophobic) substrates with bromide salt and H_2_O_2_. Under this condition, the disproportionation of H_2_O_2_ was prevented, allowing *trans*-dibromides preparation in high yields from electron-rich, electron-poor, and alkene-bearing protecting groups. The reaction rate was considerably enhanced by adding 0.1 mol % of PhTeTePh **36**.

Later, Liu et al. advanced the use of diaryl ditelluride as an activator of peroxides for oxidation reactions. The group reported on the immobilization of bis­(2-aminophenyl) ditelluride on poly­(ethylene glycol) methyl ether methacrylate and *N*-hydroxysuccinimide acrylate copolymer **97**. This polymeric nanoparticulated catalyst was effectively employed to oxidize bromide salts and thiols **12** (Scheme 30).^71^

**Scheme 30:**
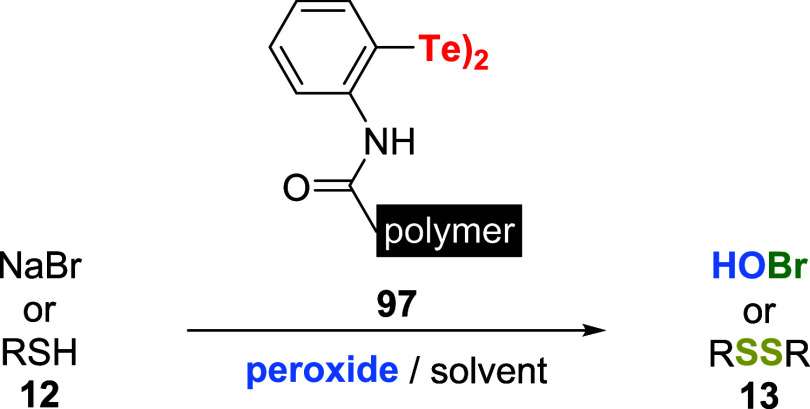
Utilization of an Immobilized Ditelluride **97** as a Catalyst for the Oxidation of Halide Salts or Thiols with H_2_O_2_

Catalyst **97** was particularly efficient for cumene hydroperoxide activation on the oxidation of thiols. On the other hand, NaBr oxidation with H_2_O_2_ was checked by UV–vis spectrophotometry following the conversion of phenol red (PR) to its brominated product (PRBr_4_). In the range of pH 4.0–9.0 buffer solutions, the best condition for oxidation was at pH 5.7 with 0.05 mg/mL of **97** (1.6 × 10^–5^ M of Te–Te). Under these conditions, approximately 94% of PR was converted to PRBr_4_ after 40 min at 25 °C. The control experiment showed a small conversion of substrate to product. Substrates such as 4-pentenoic acid **78** (R=H) and 1,3,5-trimethoxybenzene **95** were also effectively converted to brominated products.

Arai and coauthors studied the application of cyclic telluride **98**, immobilized on a polystyrene-based carboxy resin as a peroxide activator for the oxidative dimerization of thiols **12**, including polypeptides with multiple cysteinyl groups **99** (Scheme 31).^72^

**Scheme 31:**
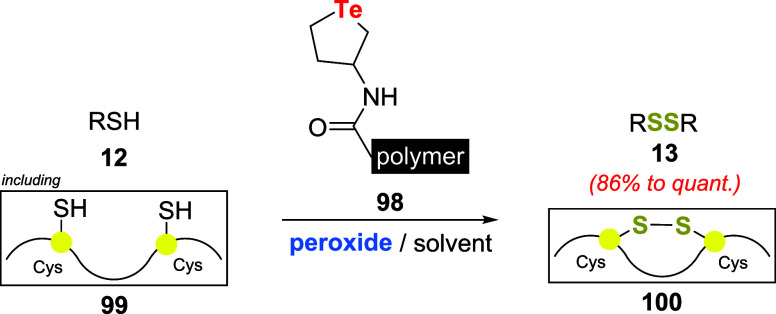
Dimerization of Thiols **12** and Polythiols **99** Using Immobilized Telluride **98** and Peroxide

Dimerization of hydrophobic thiols was achieved using 1.0 equiv of *t*-BuOOH and 0.5 mol % of **98** in CHCl_3_. Several thiols were converted to the corresponding disulfide **13** in yields varying from 86% to quantitative. No reaction acceleration was observed in control experiments (without **98**) or with the selenide analog of **98**. Remarkably, catalyst **98** was removed by suction filtration after the reaction, and pure disulfides were obtained simply by concentrating the remaining solution. Catalyst **98** could be reused, although the catalytic activity gradually decreased with an increasing number of recycles. Good catalyst activity was retained for up to three trials while maintaining the chemoselectivity on the conversion to disulfides **13**.

Hydrophilic thiols (e.g., cysteamine, glutathione, and dl-dithiothreitol) were smoothly converted to the corresponding disulfide by using H_2_O_2_ in slightly acidic aqueous solutions. Moreover, catalyst **98** promoted the activation of H_2_O_2_ for intramolecular SS bond formation in polypeptides with multiple cysteinyl SH groups **99**. This process has the potential application for oxidative folding, a fundamental chemical reaction in the industrial production of protein- and peptide-based formulations.

Koguchi et al. studied the combination of peroxides with catalytic amounts of diaryltellurium dicarboxylates **52** to promote the epoxidation of alkenes **45** (Scheme 32).^73^ Reactions using citronellyl acetate as a substrate showed that the catalytic activity of these species was affected by the nature of the aryl moiety and the carboxylate. Among the catalysts **52** evaluated, dimesityl tellurium diacetate (Ar = 2,4,6-MeC_6_H_2_) showed the best activity, while diphenyl tellurium diacetate (Ar = C_6_H_5_) or dimesityl tellurium bis­(2,2,2-trifluoroacetate) were ineffective. Additionally, the effectiveness of the epoxidation reaction was determined by the solvent and nature of the oxidizing agent. For instance, the best results were obtained using CHCl_3_ and urea hydrogen peroxide (UHP) instead of H_2_O_2_.

**Scheme 32:**
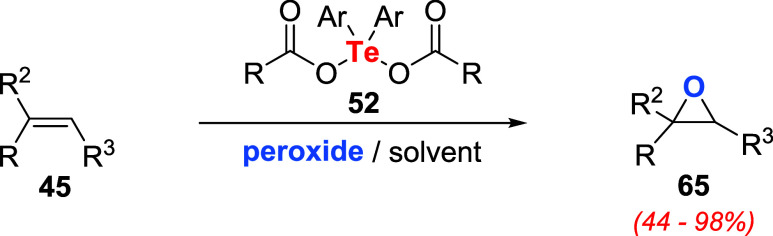
Diaryltellurium Dicarboxylates **52** as Catalysts for Epoxidations with Peroxide

Applying the best reaction conditions, **52** (10 mol %), UHP (4 equiv) in CHCl_3_ under reflux allowed the conversion of cyclic and alkyl alkenes **45** (primary, secondary, and tertiary) to the corresponding epoxide **65** in excellent yields. Terpenes were also suitable substrates for the transformation. For instance, (R)-(+)-limonene, possessing two carbon–carbon double bonds, produced the corresponding mono- and diepoxides. Diepoxides can be obtained exclusively by increasing the amount of peroxide. Cholesterol derivatives produced a mixture of two diastereomers in a 92% combined yield. On the other hand, styrene derivatives furnished the corresponding epoxides in lower yields.

Control reactions using diaryltellurium dicarboxylates **52** were designed to elucidate the reaction mechanism (Scheme 33a). It was observed that upon treatment with peroxide **52**, a mixture of benzoic acid **101** and phenol **103** was produced, presumably by the collapse of intermediate **104**. Tellurenil acetate **105**, which should be a product of this reaction, then reacted with another equivalent of UHP to produce *in situ* pertellurinic acid **94b** and **101**. The formation of the former species was confirmed by HRMS analysis. The reaction mechanism, depicted on Scheme 33b, mirrors the process observed for peracids^74^ or peroxyseleninic acids:^75–77^ pertellurinic acid **94b** reacts with the alkene **45** to deliver epoxide **65**, and tellurinic acid **90b**. The latter reacts with another peroxide equivalent to regenerate **94b** for the catalytic cycle.

**Scheme 33:**
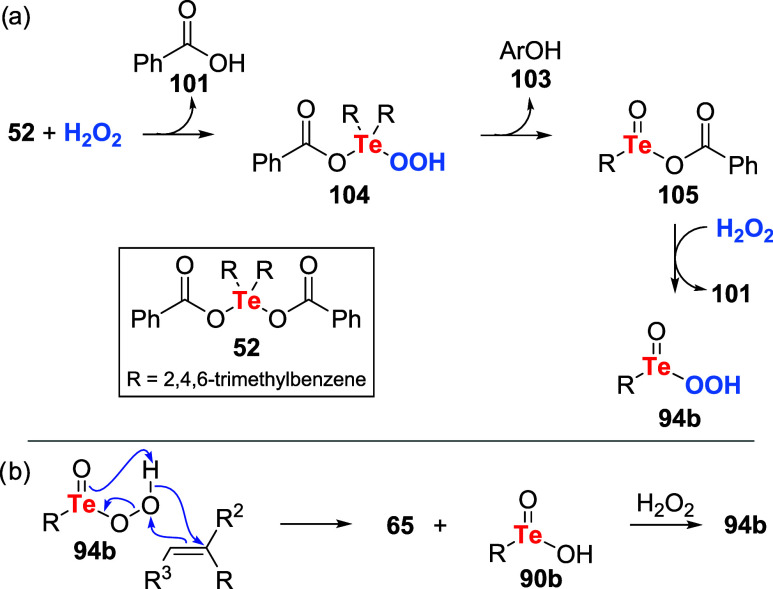
(a) *In Situ* Formation of the Active Oxidizing Agent, Pertellurinic Acid **94b**. (b) Proposed Mechanism for the Epoxidation of Alkenes with Diaryltellurium Dicarboxylates **52** and Peroxide

McKee reported the first oxidation of organic compounds using oxygen catalyzed by tellurium in 1984. He described that tellurium metal (2% in weight), its oxide, or the tetrachloride promoted the oxidation of graphite to carbon dioxide by gaseous oxygen in the temperature range of 500–800 °C (Scheme 34).^78^ Thermoanalytical data supported a reaction mechanism involving the oxidation of tellurium to tellurium dioxide (TeO_2_) by ambient oxygen, followed by its reduction to the metallic state by a reaction with the carbon substrate. Notably, the lighter chalcogens, sulfur or selenium, did not show catalytic activity.

**Scheme 34:**
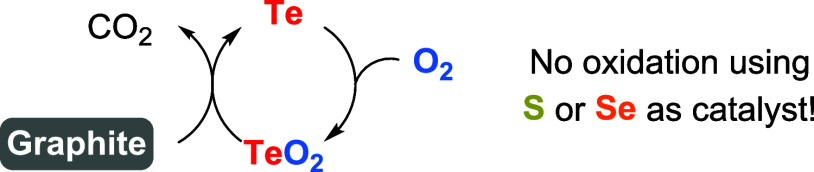
First Reported Oxidation Reaction Using Oxygen Activated by Tellurium

In 1988, Detty et al. reported the photochemical oxidation of organotellurium compounds outlined in Scheme 35.^79^ The aqueous solution of tellurapyrylium derivative **66** was irradiated with light from a tungsten lamp to obtain tellurium oxide (as hydrate **67**). After that, several studies showed that oxygen in the air, when irradiated with light, generates singlet oxygen via the near-infrared absorbing dye compound tellurapyrylium derivative, and the tellurium atom of the tellurapyrylium derivative is then oxidized by the singlet oxygen.^80^ These reactions can generate singlet oxygen without a separate sensitizer because the tellurapyrylium derivative is a dye. However, this reaction has not been used for oxidizing organic compounds.

**Scheme 35:**
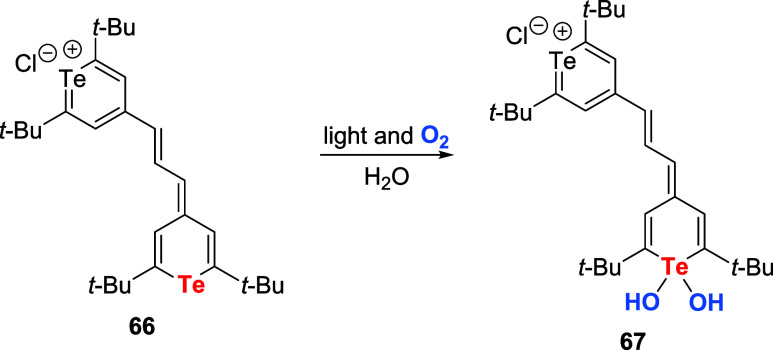
First Observation of Photooxidation of Organotellurium Compounds with Oxygen

Subsequently, the group prepared tellurorhodamine dye **106** as a self-sensitizing species and applied it as a catalyst for the aerobic oxidation of thiols **12** (Scheme 36).^81^ Oxidations of substrates were completed after 2 h of irradiation with visible light under atmospheric conditions. Oxidations of electron-poor, sterically bulky, or aliphatic thiols were slower. Notably, reactions using the corresponding selenorhodamine derivative as the catalyst resulted in a substantial decrease in the level of product formation. Two years later, the oxidation of silanes and phosphines using tellurorhodamine **106** under mild aerobic conditions was reported.^82^

**Scheme 36:**
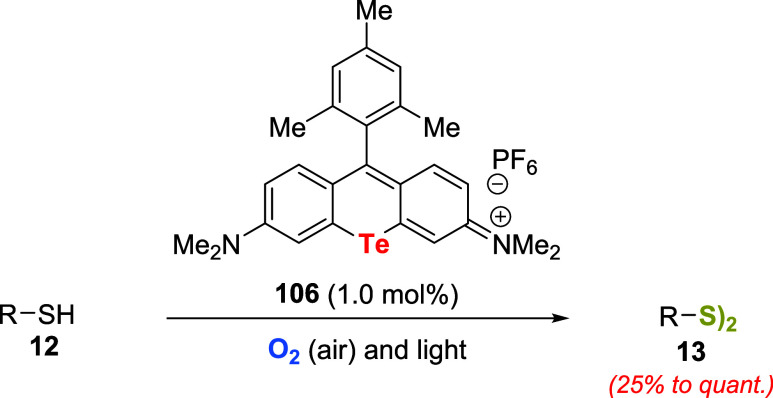
Aerobic Oxidation of Thiols **12** Using 1.0 mol % of Tellurorhodamine Dye **106**

They further used the chalcogenorhodamine derivative **106** or **107a**–**c** in the photocatalytic oxidation of 2-aryl-1,2,3,4-tetrahydroisoquinolines **108** (Scheme 37).^83^ Product selectivity was modulated by the mixture of solvents used. In MeCN/MeNO_2_ aza-Henry reaction took place to deliver products **109**. On the other hand, reactions conducted in a MeCN/water mixture gave the corresponding quinolones **110**. In both cases, products were achieved upon LED irradiation of the substrate under an air atmosphere, and only 1.0 mol % of catalysts **106** or **107a**–**c**. These photocatalytic transformations were more efficient with the selenol- and telluro derivatives **107c** and **106**, respectively. No catalytic activity was observed for thiorosamine **107b** and rosamine **107a** photocatalysts. Additionally, yields were greatly reduced when the reactions were performed under a nitrogen atmosphere.

**Scheme 37:**
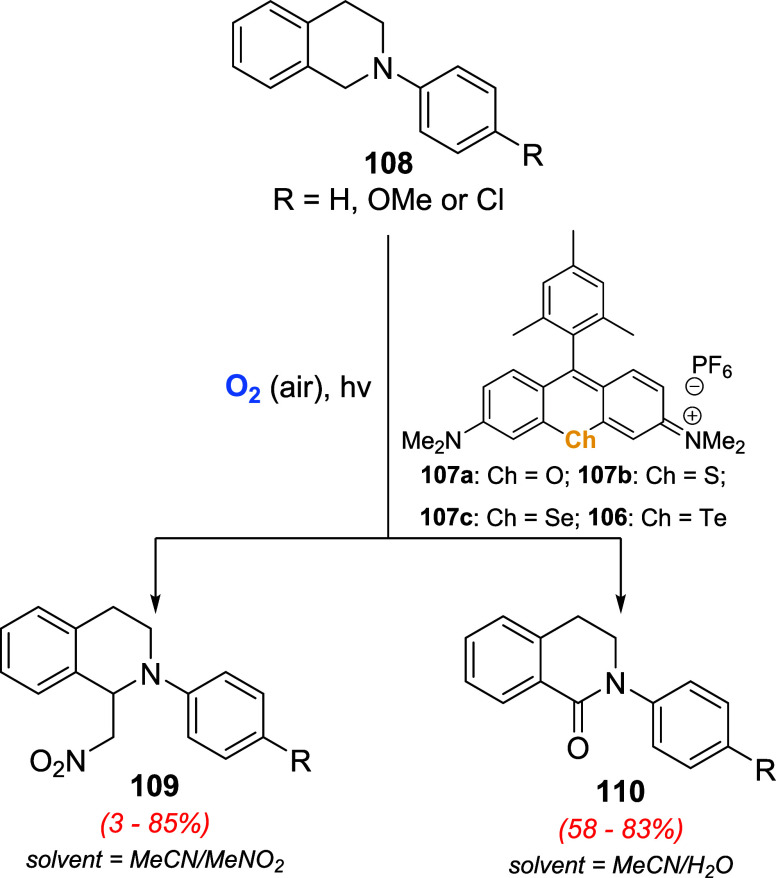
Chalcogenorhodamines **106** or **107a**–**c** in the Photocatalytic Oxidation of Tetrahydroisoquinolines **108** under Different Reaction Conditions

McCormick and co-workers further investigated the photooxidation efficiency of self-sensitized tellurorhodamine dyes. For this task, the reduction of telluroxides assembled with different electron-donating groups **111a**–**d** to the corresponding tellurides **112a**–**d** was followed under pseudo-first-order conditions (using 100 equiv of silane **113**), Scheme 38.^84^ It was found that at low temperatures (10–20 °C), the rate of formation of the oxidized product **114** correlates well with the electron-donating nature of the xylene substituent (**111d** > **111c** > **111b** > **111a**).

**Scheme 38:**
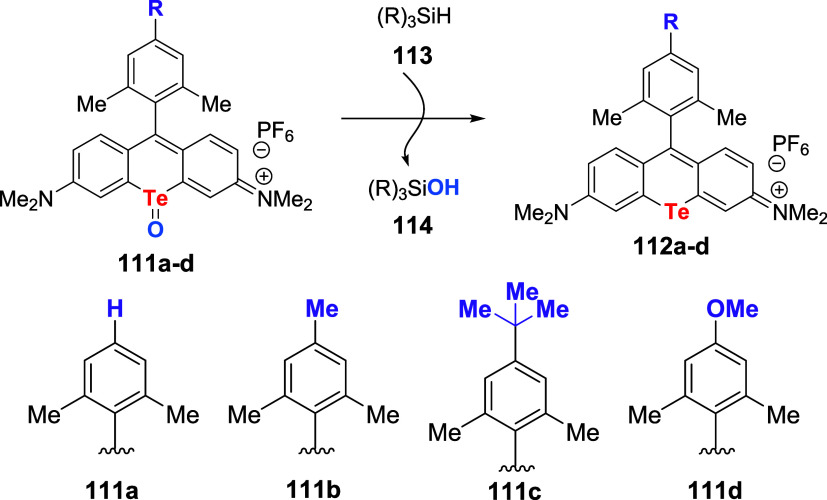
Effect of Electron-Donating Groups on the Photooxidation Efficiency of Tellurorhodamine Dyes

After almost two decades, Ando, Nishiyama, and coauthors disclosed for the first time the oxidation of simple organotellurium compounds to telluroxides using photosensitizers. The telluroxides were used in stoichiometric amounts to oxidize alcohols to the corresponding carbonyl derivative.^47^ Later, the same group reported the first application of organotellurium compounds as catalysts for photooxidation using molecular oxygen as a terminal oxidant (Scheme 39a).^85^ Dilute solutions of triphenyl phosphite **115** in acetonitrile containing 1.0 mol % of diaryl tellurides produced triphenyl phosphate **116** in quantitative yields. The best results were observed when the reaction mixture was irradiated under aerobic conditions with a 500-W halogen lamp employing Rose Bengal (RB, 10^–4^ M) as a photosensitizer and bis­(2,4,6-triisopropylphenyl) telluride **117** as the catalyst. The proposed reaction mechanism involves the conversion of oxygen to singlet oxygen by light and a sensitizer. Under these conditions, diaryl telluride **117** is oxidized to the corresponding telluroxide **49b**, which promotes product formation and regenerates the telluride **117** to the catalytic cycle (Scheme 39b).

**Scheme 39:**
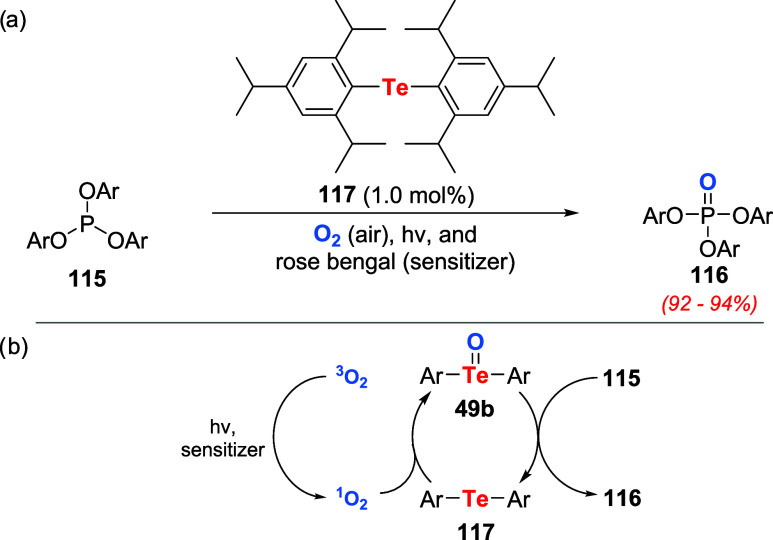
(a) Aerobic Photooxidation of Triphenyl Phosphite **115** Using 1.0 mol % of Telluride **117**. (b) Proposed Mechanism

Following these reports, the same group reported the aerobic oxidation of silanes **113** ^86^ and thiols **12** ^87^ to silanols **114** and disulfides **13** under photosensitized conditions using organotellurium catalysts (Scheme 40). In the case of silanes, a pyridine solution of silane **113**, 10.0 mol % of bis­(2,4,6-trimethylphenyl) telluride **118**, and hematoporphyrin (10^–4^ M) as the photosensitizer in an open flask was stirred vigorously and irradiated with a 500-W halogen lamp for 4 h. This condition resulted in the quantitative production of the corresponding silanols **114**. Similarly, a dichloromethane solution of the thiol **12**, 1.0 mol % of bis­(4-methoxyphenyl) telluride **19**, and tetraphenylporphyrin (TPP, 0.1 mM), used as the photosensitizer, was stirred vigorously in an open flask and irradiated with a 500-W halogen lamp at 0 °C to give the corresponding disulfides **13** in good to excellent yields.

**Scheme 40:**
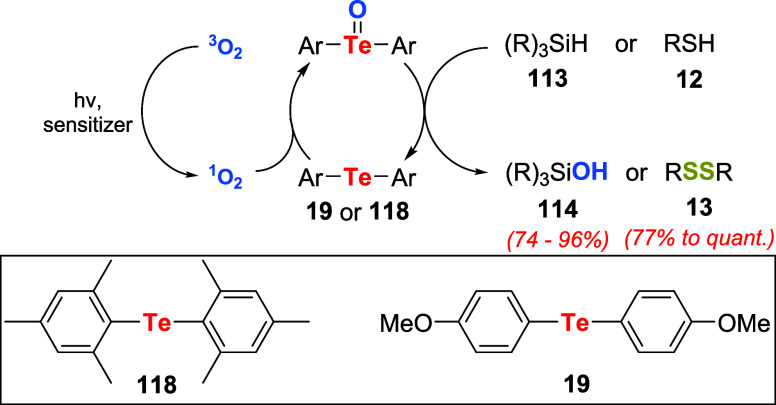
Aerobic Photooxidation of Silanes **113** or Thiols **12** Using Organotellurium Catalysts

Oba, Nishiyama, Ando et al. further investigated the aerobic oxygenation of diaryl tellurides **119a**–**d** under photosensitized conditions.^88^ They observed that the reaction of these compounds with singlet oxygen typically results in the smooth formation of telluroxides **120a**–**d** and tellurones **121a**–**d** (Scheme 41). Product distribution was significantly influenced by the substrate’s nature and the reaction conditions. When Ph_2_Te **119a**, An_2_Te **119b**, and Mes_2_Te **119c** were photo-oxidized in a protic solvent such as ethanol, the corresponding telluroxides **120a**–**c** were selectively produced in quantitative yields. On the other hand, under the same conditions, the bulkier diaryl telluride Tip_2_Te **119d** produced a 1:1 mixture of telluroxide **120d** and tellurone **121d**. For this telluride, reactions at low temperatures and under diluted conditions in aprotic solvents, such as dichloromethane, acetonitrile, or pyridine, resulted in increased amounts of tellurone **121d**.

**Scheme 41:**
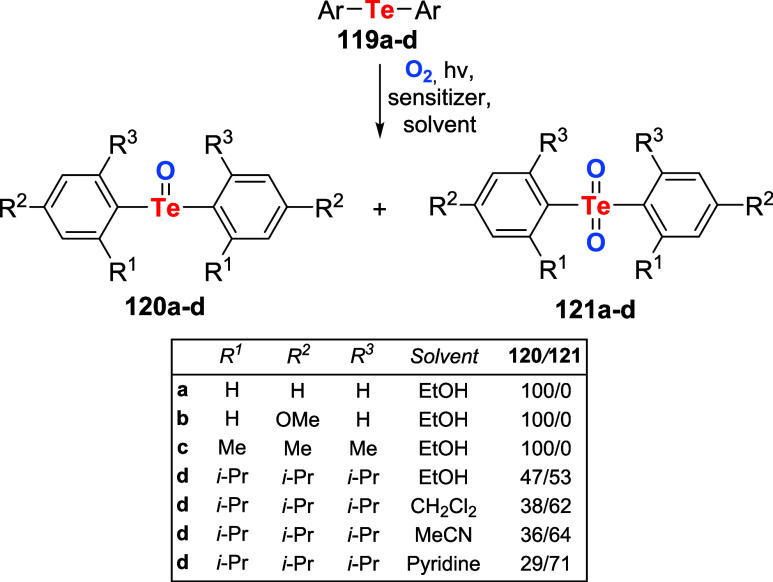
Aerobic Oxidation of Diaryl Tellurides

Recently, Koguchi et al. prepared ionic-liquid-supported organotellurium compounds **122**. These compounds effectively promoted thiols **12** ^89^ and phosphite esters **115** ^90^ oxidations under photosensitized oxygenation conditions with a suitable photosensitizer in ionic liquids (Scheme 42). The reaction media (catalyst, photosensitizer, and ionic liquid used as solvent) could be recycled and reused efficiently several times.

**Scheme 42:**
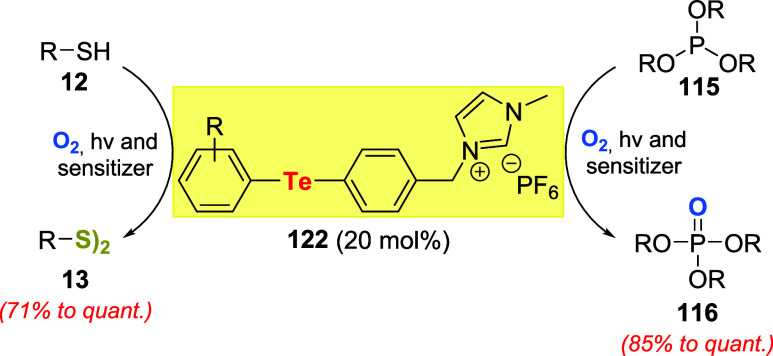
Use of an Ionic Liquid-Supported Telluride **122** for Aerobic Photooxidation of Organic Substrates

Afterward, Wolf and coauthors used diphenyltelluride to promote the oxidation of sulfides on polydimethylsiloxane copolymers **123**. The interest in the disulfide linkage on polymeric material **124** is attractive for the preparation of degradable materials. To achieve the task, 1 weight% of diphenyltelluride, relative to **123**, was excited in the presence of oxygen, blue light, and tetraphenylporphyrin (TPP) and the photosensitizer (Scheme 43).^91^

**Scheme 43:**
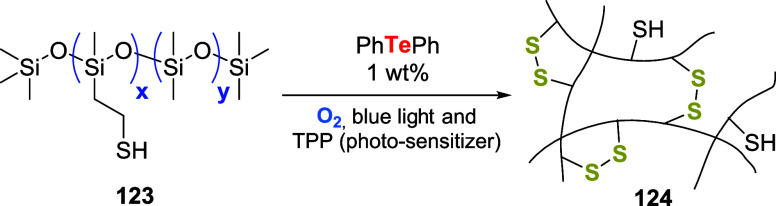
Photo-Oxidative Formation of Disulfide Bonds in Polymers Catalyzed by PhTePh

The mechanism for the catalytically active species’ formation in the oxidations mentioned above using oxygen is depicted in Scheme 44. It is very similar to the previously known oxidation of sulfides under aerobic conditions.^92–97^ Initially, upon irradiation with light and a photosensitizer, triplet oxygen is converted to singlet oxygen, which is quenched by telluride **8**, producing pertelluroxide intermediate **125**. When the reaction occurs in water or alcohol, **125** rapidly interacts with the solvent, forming hydroperoxytellurane **126**. Finally, **125** or **126** reacts with the remaining **8** to produce two equivalents of telluroxide **84**. Occasionally, telluroxide **84** can react with another equivalent of **125** or **126**, producing a mixture of tellurone **127** and **84**. In the case of tellurium compounds with bulky substituents, such as Tip_2_Te **119d**, solvent addition might be significantly hampered due to steric hindrance. On the other hand, in aprotic solvents, the main product of the reaction between telluride **8** and oxygen is tellurone **127**. It is reasoned that in this scenario, product formation is preceded by the formation of the dioxatellurirane **128** intermediate or the corresponding dimer **129**.^79–81,83,88,98,99^

**Scheme 44:**
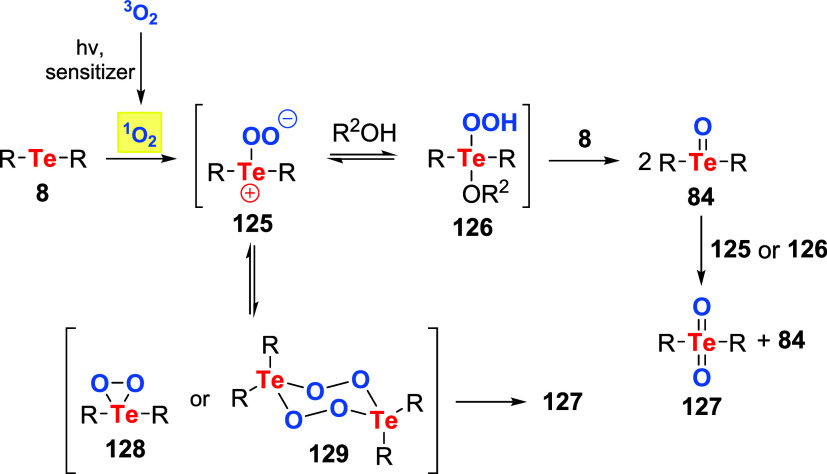
Observed Products of Telluride **8** Aerobic Photooxidation

In 2019, Zhou and Yu introduced the utilization of commercially available PhTeTePh **36** as a catalyst for the oxidative deoxidation reaction of oximes **130** to ketones **131** (Scheme 45).^100^ Optimized reaction conditions were found using 2.5 mol % of **36** and oxygen as the oxidizing agent (O_2_ balloon) under solvent-free conditions and heating.

**Scheme 45:**
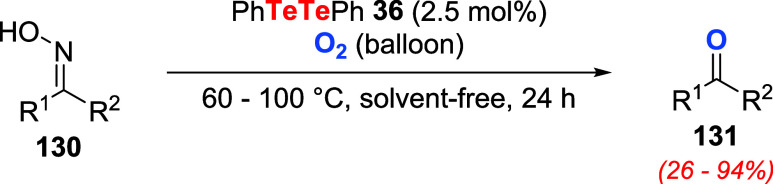
Aerobic Oxidation of Oximes **130** Utilizing PhTeTePh **36** as a Catalyst

Several oximes could be converted to the corresponding ketones under these conditions. It was observed that cyclic oximes were inert and that substrates bearing long alkyl chains, strong electron-donating groups, or bulk alkyl groups were more resistant toward the transformation. On the other hand, after product separation by vacuum distillation, the catalyst could be recycled and reused up to three times, maintaining the reaction efficiency. Control experiments indicated that the reaction proceeded through a free-radical mechanism (reaction slowed under a nitrogen atmosphere or with a radical scavenger such as TEMPO and accelerated with a radical initiator such as AIBN). X-ray photoelectron spectroscopy (XPS) analysis indicated that under oxygen and heating, PhTeTePh produced initially a mixture of Te­(II) and Te­(IV) species, presumably **132** and **133**, respectively. After 24 h of reaction, only Te­(IV) **133** species could be detected.

The proposed reaction mechanism starts with the oxidation of PhTeTePh to Te­(II) intermediate **132** (Scheme 46). Under heat and excess oxygen, this species is further oxidized to Te­(IV) derivative **133**. The addition of **133** to substrate **130** results in intermediate **134**, which then decomposes to products **131** and **135**, which are converted to **133** to resume the catalytic cycle. Two years later, the same group disclosed that under visible light irradiation, the reaction proceeded smoothly. Products were obtained upon blue light LED irradiation under solvent-free conditions using as little as 1.0 mol % % of PhTeTePh in an oxygen atmosphere.^101^

**Scheme 46:**
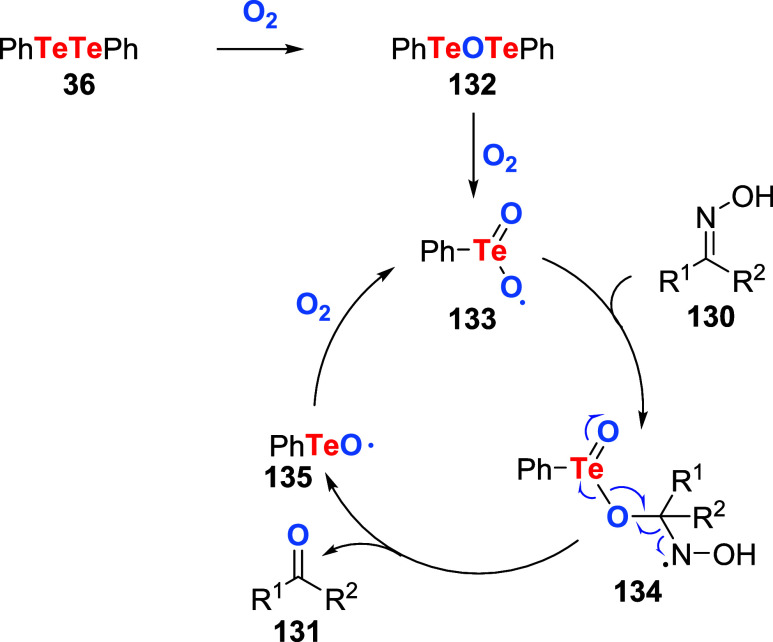
Proposed Mechanism for the Aerobic Oxidation of Oximes **130** Utilizing PhTeTePh **36** as a Catalyst

Patureau and co-workers disclosed another example of the application of organotellurium compounds to catalyze oxidation reactions. They reported the utilization of phenotellurazine **136a** as an oxygen activator for cross-dehydrogenative coupling (CDC) reactions.^102^ The model reaction explored was an O_2_-mediated cross-dehydrogenative phenothiazination of phenols **137** with phenothiazine derivatives **138** under basic conditions (Scheme 47).

**Scheme 47:**
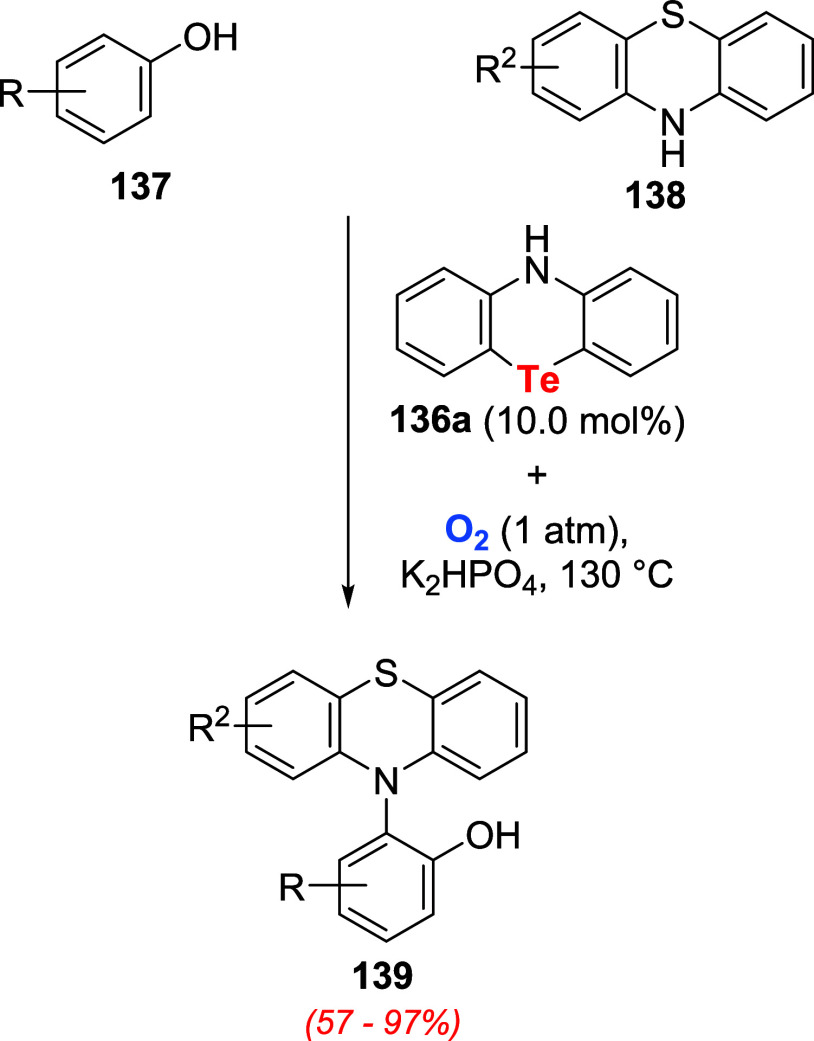
Cross-Dehydrogenative Phenothiazination of Phenols **137** with Phenothiazine Derivatives **138** under Aerobic Conditions Catalyzed by Telluride **136a**

The substrate scope was broad, including phenothiazine derivative **138** assembled with electron-donating and electron-withdrawing groups. Most importantly, a wide panel of phenols **137**, including more challenging electron-poor and derivatives with pendant functional groups such as ketone, pyridine, and aldehyde, were suitable for the transformation. The proposed reaction mechanism was corroborated by a series of control experiments, including kinetic experiments, cyclic voltammetry (CV), electron paramagnetic resonance (EPR) spectroscopy, and density functional theory (DFT) calculations utilizing the four chalcogen congeners (**136b**–**d**) of catalyst **136a**. The mechanism is initiated by the oxidation of Te­(II) species **136a** to the corresponding Te­(III) radical cation species **140a** (Scheme 48). Deprotonation of **140a** by the basic media delivers neutral radical **141a**, which in an H-atom transfer (HAT) process with substrate **138** produces persistent radical **142** and regenerates Te­(II) catalyst **136a**. Species **142** reacts with phenols to deliver product **139**.

**Scheme 48:**
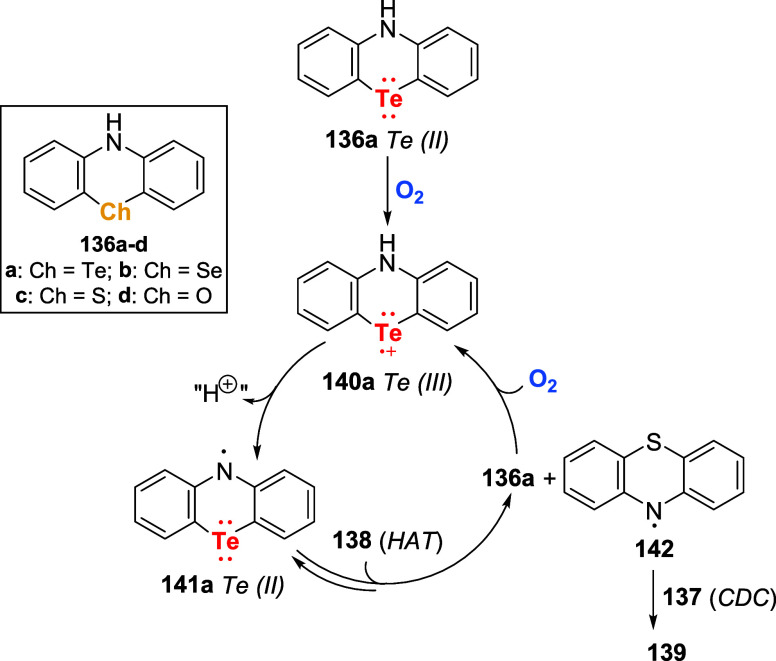
Proposed Mechanism for the Cross-Dehydrogenative Phenothiazination of Phenols **137** with Phenothiazine Derivatives **138** under Aerobic Conditions Catalyzed by Telluride **136a**

Control experiments with **136a**–**d** suggested that the catalytic effect of **136a** stems from the combination of the lower oxidation potential of **136a** (+0.08 V vs +0.24 for O and Se and +0.22 for S) and the significantly higher spin density at the tellurium center compared to the other chalcogen-based catalysts and substrates. Combined, these properties allowed the formation of **140a** and, subsequently, **141a**, which reacts by a HAT process with **138** faster than by CDC with phenols **137**.

In the following years, the same group investigated the utilization of a broader library of tellurium catalysts for cross-dehydrogenative coupling (CDC) of phenols **137** with phenothiazine derivatives **138** using oxygen as the oxidant^103^ and applied these catalysts to a broader scope of CDC reactions. For instance, aniline derivatives **143** were smoothly converted to densely functionalized products **144** (Scheme 49). The reaction is accelerated by adding 10.0 mol % of telluride **136a**, and the best oxidizing agent screened was silver oxide (Ag_2_O).^104^ The developed process allowed the conversion of several anilines and diarylamines tethered with various functional groups to the desired products.

**Scheme 49:**
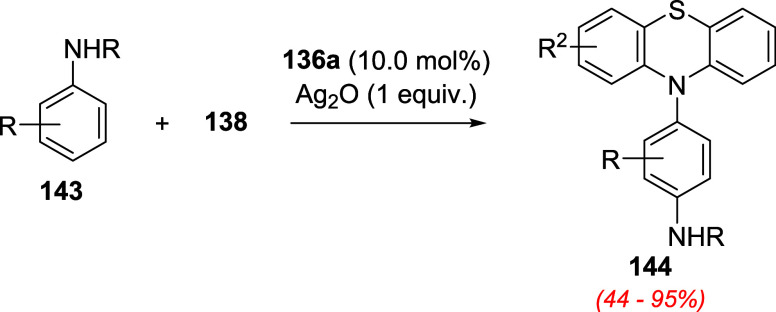
Cross-Dehydrogenative Coupling of Anilines **143** with Phenothiazine, Ag_2_O, and Telluride **136a**

The developed Te­(II)/Te­(III) redox catalysis concept was further investigated for the dehydrogenative C3–C2 dimerization of indoles **145** to **146**. The catalytic performance of tellurides **147** increased with the electron-releasing power of substituent R: **147c** > **147a** > **147b**. Using 10.0 mol % of telluride **147c**, several indols **145** were oxidized to the respective dimer **146** using oxygen as the terminal oxidizing agent (Scheme 50).^105^

**Scheme 50:**
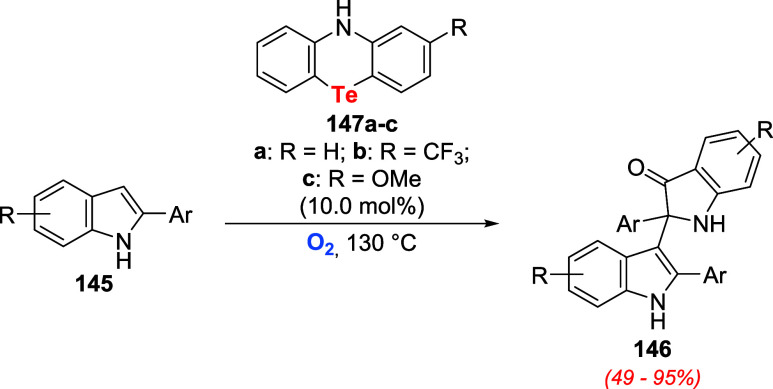
Aerobic Dimerization of Indols **145** Promoted by Tellurides **147a**–**c**

## Conclusions

Considering the pivotal importance of oxidation reactions in different fields, research on this topic is constantly evolving. In this review, we discuss the application of organotellurium compounds for such chemical transformations. Initial efforts in this scenario were based on organotellurium compounds as stoichiometric oxidants. Several groups have shown that reactions can deliver various products efficiently and selectively. Major drawbacks of the process were the necessity to oxidize the organotellurium compound to an active form previously, usually telluroxides or tellurones, and the formation of organotellurides as side products of the reaction.

Significant progress has been achieved in organotellurium compounds for oxidation reactions. Noteworthy was the discovery that these compounds could be used in catalytic amounts along with oxidizing agents. It paved the way for achieving more environmentally friendly protocols to affect the conversion of several substrates to desired products. Various research groups have devised catalysts tethered to solid supports, allowing for the recycling and reuse of these species. Additionally, the utilization of oxidizing agents such as peroxides and, ultimately, molecular oxygen and light rendered such transformation even more appealing in an environmental scenario.
